# Synergistic promotions between CO_2_ capture and in-situ conversion on Ni-CaO composite catalyst

**DOI:** 10.1038/s41467-023-36646-2

**Published:** 2023-02-22

**Authors:** Bin Shao, Zhi-Qiang Wang, Xue-Qing Gong, Honglai Liu, Feng Qian, P. Hu, Jun Hu

**Affiliations:** 1 Key Laboratory for Advanced Materials and Joint International Research Laboratory for Precision Chemistry and Molecular Engineering, Feringa Nobel Prize Scientist Joint Research Center, Centre for Computational Chemistry and Research Institute of Industrial Catalysis, School of Chemistry and Molecular Engineering, East China University of Science and Technology, 130 Meilong Road, Shanghai, 200237 China; 2 State Key Laboratory of Chemical Engineering, School of Chemical Engineering, East China University of Science and Technology, 130 Meilong Road, Shanghai, 200237 China; 3 Key Laboratory of Advanced Control and Optimization for Chemical Processes of Ministry of Education, School of Information Science and Engineering, East China University of Science and Technology, 130 Meilong Road, Shanghai, 200237 China; 4 School of Chemistry and Chemical Engineering, The Queen’s University of Belfast, Belfast, BT9 5AG UK

**Keywords:** Heterogeneous catalysis, Catalytic mechanisms, Computational chemistry, Density functional theory, Pollution remediation

## Abstract

The integrated CO_2_ capture and conversion (iCCC) technology has been booming as a promising cost-effective approach for Carbon Neutrality. However, the lack of the long-sought molecular consensus about the synergistic effect between the adsorption and in-situ catalytic reaction hinders its development. Herein, we illustrate the synergistic promotions between CO_2_ capture and in-situ conversion through constructing the consecutive high-temperature Calcium-looping and dry reforming of methane processes. With systematic experimental measurements and density functional theory calculations, we reveal that the pathways of the reduction of carbonate and the dehydrogenation of CH_4_ can be interactively facilitated by the participation of the intermediates produced in each process on the supported Ni–CaO composite catalyst. Specifically, the adsorptive/catalytic interface, which is controlled by balancing the loading density and size of Ni nanoparticles on porous CaO, plays an essential role in the ultra-high CO_2_ and CH_4_ conversions of 96.5% and 96.0% at 650 °C, respectively.

## Abstract (web summary)

The integrated CO_2_ capture and conversion (iCCC) technology has been booming for carbon neutrality. Here the authors optimized the Ni–CaO composite catalyst to promote iCCC involving consecutive high-temperature Calcium-looping and dry reforming of methane and illustrated their synergistic promotions at the suitable catalyst interface.

## Introduction

Carbon capture, utilization and storage (CCUS) technology can play a crucial role to restrain the global warming of no more than 1.5 °C above pre-industrial levels^1,2^. Among various CCUS technologies, the integrated CO_2_ capture and conversion (iCCC) has attracted more and more interests since it shows great advantages in saving energies and costs for CO_2_ compression and transportation involved in the conventional CCUS processes^3–5^. More significantly, when the iCCC technology is applied to the CO_2_ capture from the high-temperature flue gas, its thermo-energy can be directly converted into the chemical energy during CO_2_ conversion, resulting in high energy efficiency^6,7^.

Intuitively, the successful application of iCCC lies in the development of dual-functional materials (DFMs), of which the adsorptive sites and catalytic sites are intimately close to each other^8^. Then, the synergistic promotion effects between the CO_2_ capture and in situ conversion may result in both high and stable CO_2_ capture capacity and conversion efficiency^9,10^. Currently, the reported DFMs in most iCCC processes are CaO-based composites largely due to its excellent theoretical CO_2_ capacity (17.8 mmol g^−1^) at high temperatures^11,12^. Instead of the calcination regeneration at above 900 °C in the traditional Calcium looping (CaL)^13,14^, the in situ conversion of captured CO_2_ may significantly lower the regeneration temperature and thus overcome the bottleneck problems of high energy penalties and CaO sintering. Meanwhile, catalysts such as Rh^15^, Ru^16^, Ni^17^, Fe^18^, and Co^19^ in C1 chemistry of methanation, reverse water gas shift reaction (RWGS), and dry reforming of methane (DRM), are usually combined within iCCC processes for the CO_2_ conversion. However, the development of DFMs has underlined the gap in mechanism investigation, that the interactions and synergy effects between sorbents and catalysts of DFMs need significant molecular-level understanding to break through the efficiency limitation of DFMs^20^. So far, even the nature of the active sites in DFMs and its role in the conversion of captured CO_2_ are still under debate, and it is not clear if the adsorption site can solely accommodate the CO_2_ and its catalytic conversion can only occur at other positions^21–23^, or the captured CO_2_ can be also activated at the adsorption site for direct reaction with the incoming H species produced on the nearby catalytic sites^24–27^. Therefore, how the synergistic effects between the adsorptive and catalytic sites may work at the DFMs interface and what types of elementary steps are involved in the consecutive reaction pathways need intensive research for the development of efficient iCCC strategy^20,28^.

At the same time, the dry reforming of methane (DRM) is a very useful reaction, which can convert two major greenhouse gases of CO_2_ and CH_4_ into valuable syngas with equimolar H_2_ and CO. The challenges in the development of DRM mainly come from the high energy consumption due to the strong endothermic nature of this reaction and the corresponding high-temperature (>800 °C)^29^. Moreover, it also suffers from the catalyst deactivation caused by the coking^30^. Currently, most studies focus on developing efficient DRM catalysts for the activation of the two reactants, while the strategies to take advantage of their coupled reactions are still limited.

Herein, we chose the most representative high-temperature iCCC technology by integrating the Calcium-looping and dry reforming of methane (CaL@DRM) processes. For the first time, we demonstrated the synergistic promotion effects between CO_2_ capture and in-situ conversion on the Ni–CaO DFM in a molecular way. Density functional theory (DFT) calculations of the corresponding model catalyst with a Ni_4_ cluster on the CaO(100) substrate disclosed the synergistic reaction pathways of the direct conversion of CaCO_3_ by the DRM, which settles the debate about the role of captured CO_2_ on conversion. More importantly, the mechanism of synergistic promotions provided the in-depth understanding of the long-sought consensus about the consecutive reactions involved in CaL@DRM and inspired the design and fabrication of DFMs with enriched interface between the catalytic site and adsorptive site. With the controlled loading density and the growth of Ni nanoparticles in the nanoconfined space of porous CaO substrate, such optimized Ni–CaO DFMs can give rise to high conversion efficiencies for both CO_2_ and CH_4_, superior to all the reported performance of conventional catalysts and DFMs for the DRM process. We anticipate that the findings will help to build an efficient way to boost the iCCC technology for Carbon Neutrality, and to shine the light on the general understanding of consecutive reactions.

## Results

**Fig. 1:**
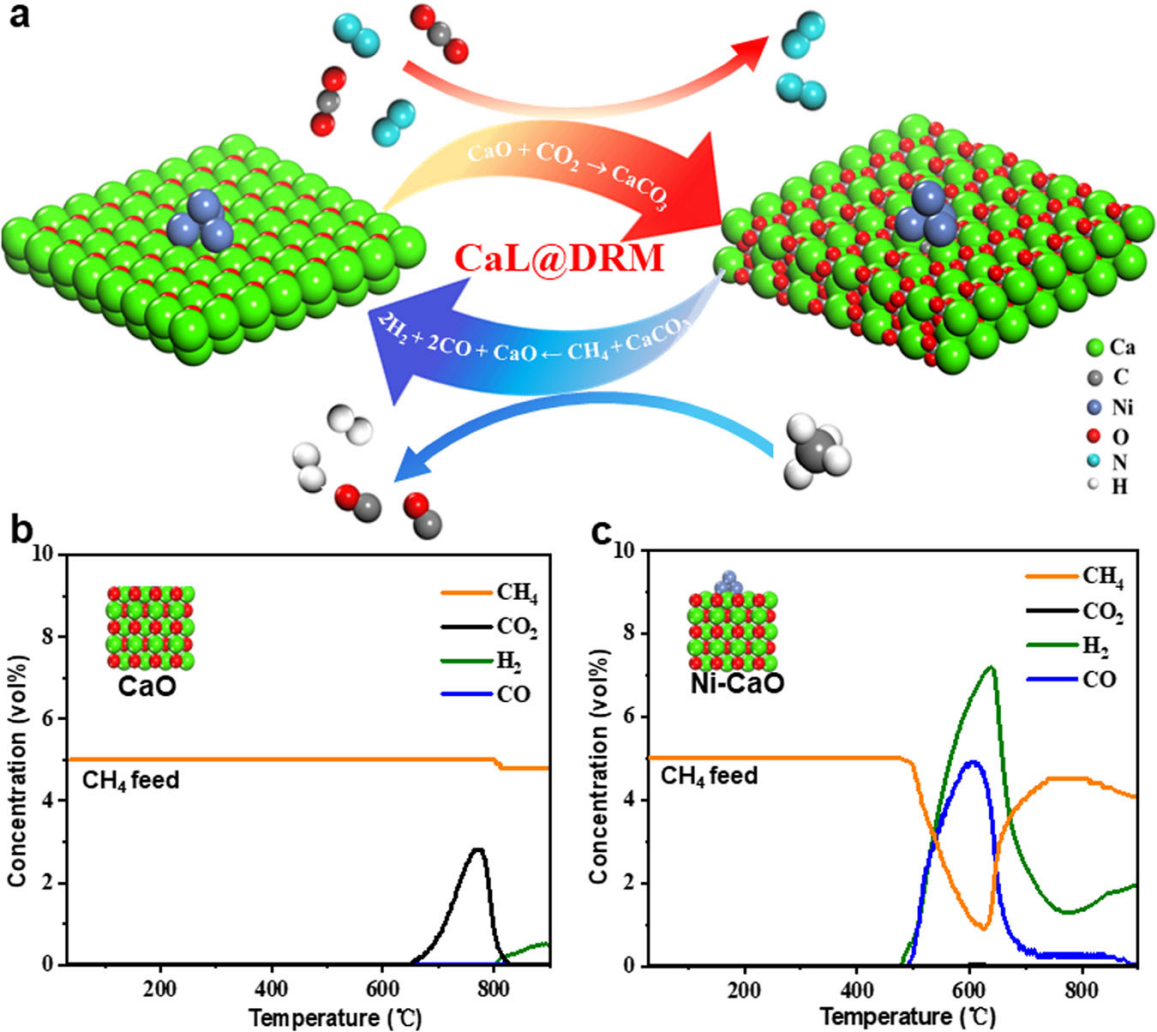
CaL@DRM on the Ni–CaO dual-functional material. **a** Schematic illustration of the proposed CaL@DRM iCCC processes. The performances of the CH_4_ temperature-programmed surface reactions (TPSR) on the **b** CaO and **c** Ni–CaO-10 DFM after CO_2_ pre-adsorption.

The most convenient approach for the construction of possible CaL@DRM iCCC catalysts is to combine the adsorptive sites (CaO) and the catalytic sites (Ni) in one DFM (Fig. 1a). So, we intuitively fabricated a Ni–CaO-10 (Ni–CaO-*x*, *x* denotes the loading weight percentage of Ni in sample, see Supplementary Information) DFM with Ni-rich loading considering that the activation of CH_4_ could be particularly difficult in the in situ CO_2_ conversion by DRM. The temperature programed surface reaction (TPSR) was first used to evaluate the CaL CO_2_ capture process, CH_4_ dehydrogenation and CaL@DRM processes on pure CaO and Ni–CaO-10 DFM, respectively. For the CO_2_ capture from the simulated flue gas through the CaL process, Ni–CaO-10 exhibited similar CO_2_-TPSR performances as CaO, giving a downward CO_2_ adsorption peak centered at 650 °C (carbonation) and an upward CO_2_ release peak at 850 °C (decarbonization) (Supplementary Fig. 1a, 1b)^31^, demonstrating the negligible influence of supported Ni on CaL process. As the thermal cracking of CH_4_ (CH_4_ → C + 2H_2_, ∆*H*_298K_ = +74.6 kJ/mol) is an endothermic reaction, it will be more favorable to occur under higher temperature. Thus, the thermal cracking of CH_4_ was determined in this work when the temperature was high enough even without the help of any catalyst, giving rise to the CH_4_–TPSR curves of the pure CaO sample and the CaO after CO_2_ pre-adsorption (CaCO_3_), together with the H_2_ formation, above 800 °C (Fig. 1b and Supplementary Fig. 1c). Whereas the as-formed CaCO_3_ caused by CO_2_ pre-adsorption only showed a CO_2_ desorption peak at a relatively lower temperature of 780 °C. These results demonstrate the inertness of the CaO for the DRM reaction. By contrast, the CH_4_-TPSR measurement of the Ni–CaO-10 with pre-adsorbed CO_2_ showed that both CH_4_ consumption and H_2_ production started at 485 °C (Fig. 1c), which is the same as that during the CH_4_ dehydrogenation on the clean Ni–CaO-10 (Supplementary Fig. 1d), suggesting that the supported Ni should be the catalytically active site for CaL@DRM. In addition, the CO peak at the temperature above 500 °C can be also seen, and this slightly higher temperature over that of CH_4_ dehydrogenation suggests the produced surface H species (*H) may even participate in the reduction of the adsorbed CO_2_ at CaO^19,32^. Moreover, since no obvious CO_2_ signal was observed, we may suggest that the captured CO_2_ (CaCO_3_) can be indeed effectively reduced by CH_4_ to produce syngas. It is also worth mentioning that the peak values of CO and H_2_ are centered at 600–650 °C, much lower than the reported operating temperatures of 700–800 °C of the conventional DRM^33^, demonstrating the significant promotions between CO_2_ capture at CaO and its in-situ conversion through DRM. However, we also found that after the CaL@DRM processes, the Ni–CaO-10 lost its silver-like metallic luster and appeared black in color, suggesting that the bottleneck problem of the carbon deposition in the conventional DRM may still exist^29^. Therefore, we highly anticipated that a better understanding of how the CO_2_ adsorption and DRM conversion occur in an interactive way in our CaL@DRM processes would help guide further optimization of the Ni–CaO dual-functional materials.

### Mechanism for CaL@DRM process on Ni–CaO DFM

**Fig. 2:**
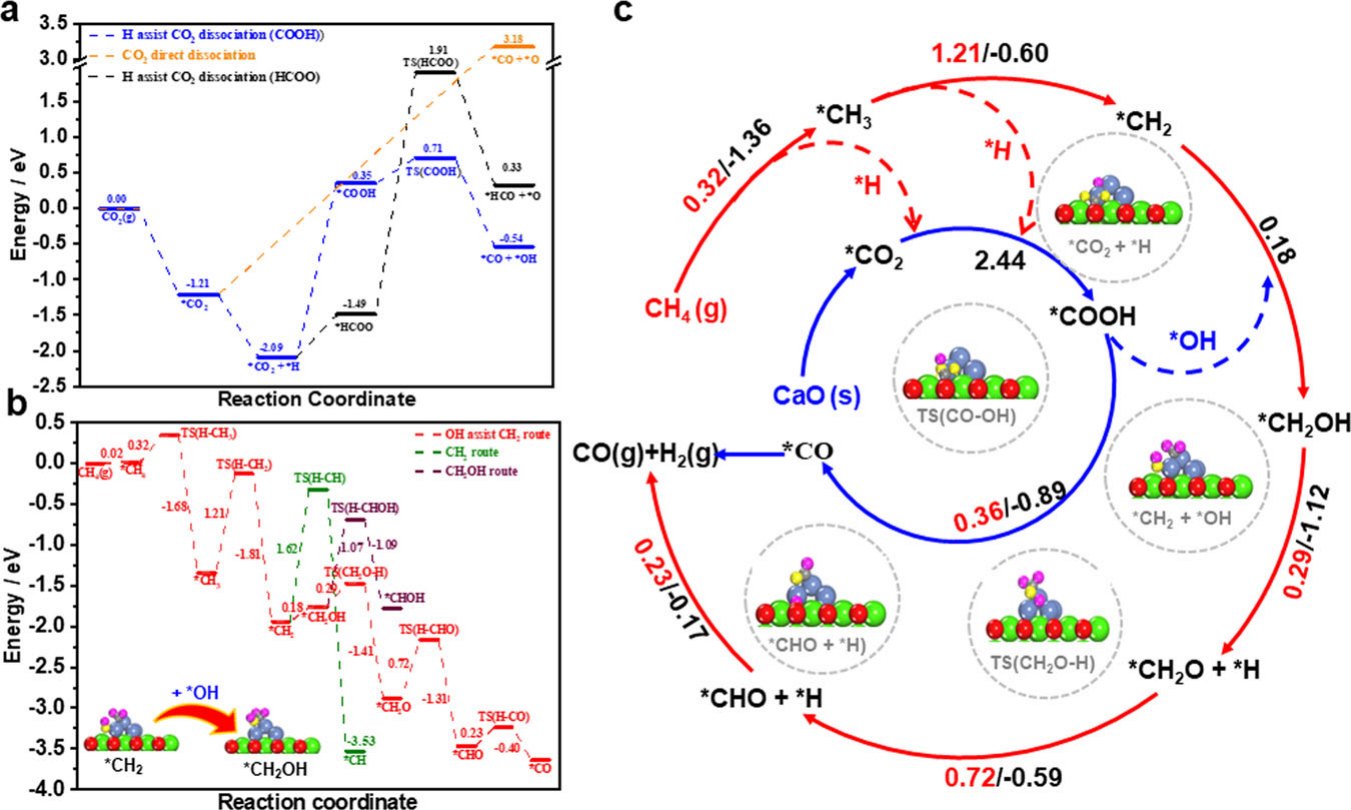
DFT calculations and proposed reaction mechanism for CaL@DRM. **a** Calculated energy profiles of CO_2_ adsorption and dissociation on the Ni_4_–CaO(100) surface; the black dashed line represents the *H-assistant CO_2_ dissociation of *HCOO, the orange dashed line represent the direct CO_2_ dissociation to *CO, and the blue dashed line represents the *H-assistant *COOH pathway. **b** Calculated energy profiles of CH_4_ dehydrogenation with the assistance of *OH species on the Ni_4_–CaO(100) surface (red dashed line); the green dashed line represents the *CH_2_ dissociates to *CH, and the brown dashed line represents the *CH_2_OH dissociates to *CHOH. **c** Schematic illustration of reaction network for the CaL@DRM on the Ni_4_–CaO(100) surface. The corresponding activation energy *E*_a_ (red) and reaction energy (black) in the unit of eV for each step are also included. The optimized structures of reaction intermediates and transition state (TS) are shown in **b** and **c**. Red: O; green: Ca; blue: Ni; yellow: O of CO_2_; Pink: H of CH_4_. This notation is used throughout the paper.

To illuminate the molecular mechanism of the synergistic promotions, we constructed the model Ni–CaO DFM with 4-atoms Ni clusters being supported on the CaO(100) substrate, namely Ni_4_–CaO (Supplementary Fig. 2), and conducted the density functional theory (DFT) calculations. The calculated results showed that a single CO_2_ prefers to be adsorbed on the CaO surface by forming one C–O(CaO) bond and two (CO_2_)O–Ca bonds, i.e., a carbonate-like adsorbed species (*CO_2_) occurs (Supplementary Fig. 3). The calculated adsorption energy (*E*_ads_) is 1.21 eV, slightly stronger than that of the CO_2_ on the Ni_4_ cluster (*E*_ads_ = 1.19 eV), and similar results can be also obtained from supported Ni_13_ clusters (Supplementary Fig. 4)^34,35^. Moreover, the calculated adsorption energies of CO_2_ on the different sites of CaO(100) around the Ni_4_ cluster are all very close with each other, indicating the moderate mobility of the adsorbed CO_2_ on the CaO(100) surface^36,37^. Notably, the dispersion corrections by using the DFT–D2 method^38,39^ and Hubbard U correction^40^ were also considered in the calculation of Ni based materials, and the calculated results showed that the overall trend of CO_2_ adsorption at different sites of the Ni_4_–CaO(100) surface were consistent with that obtained without Grimme D2 or U corrections (Supplementary Fig. 5, 6). Then, the following conversion includes a complicated reaction network involving various intermediates produced by the evolution of CH_4_ and adsorbed CO_2_. For simplicity, we first calculated the energy barrier of each step of CH_4_ dehydrogenation pathways of *CH_4_→ *CH_3_ + *H → *CH_2_ + *H → *CH + *H → *C + *H on the Ni sites at Ni_4_-CaO(100), which are 0.32 eV, 1.21 eV, 1.62 eV and 2.61 eV, respectively (Supplementary Figs. 7 and 8). The produced *H species may either combine together to produce H_2_ or participate in CO_2_ conversion^41–43^. According to our calculations, *H overflow from the Ni site to the nearby *CO_2_ to form *COOH gives an energy barrier of 2.44 eV, and the as-formed *COOH can further dissociate into *CO + *OH with a small energy barrier of 0.36 eV and the overall process is exothermic by 0.89 eV (Fig. 2a and Supplementary Fig. 9). Alternatively, *H may also react with *CO_2_ to form the *HCOO species, which needs to overcome the energy barrier of 3.40 eV to dissociate into *HCO + *O, with an endothermic reaction energy of 1.82 eV. In contrast, the direct conversion of *CO_2_ to *CO + *O species on the Ni_4_–CaO(100) is quite difficult, since the calculated endothermic reaction energy is as high as 4.39 eV. From calculated energetics of the above three different pathways, it can be concluded that the *COOH pathway is more favorable for the H-assisted *CO_2_ dissociation (Fig. 2c, blue ring). These results then indicate the synergistic promotion of CaL@DRM pathway on the Ni_4_–CaO(100) surface, as the captured *CO_2_ can be readily converted with the help of *H from the CH_4_ dehydrogenation.

In addition, one may notice that in the above direct dehydrogenation of CH_4_, rather high energy barriers need to be overcome when the intermediate *CH_2_ species undergoes deeper dehydrogenation (Supplementary Fig. 7). Interestingly, according to our calculations, the *OH produced from the conversion of *CO_2_ can readily react with the *CH_*x*_ (*x* = 2, 1, 0) at the Ni sites to form *CH_x_OH (Supplementary Fig. 8, 10). It is found that *CH_2_OH dissociate to *CHOH species need to overcome energy barrier of 1.07 eV, which is 0.78 eV higher than that of *CH_2_OH dissociate to *CH_2_O species (Fig. 2b). So, the produced *OH would assist *CH_2_ oxidation through the pathway *CH_2_ + *OH → *CH_2_OH → *CH_2_O + *H → *CHO + *H → *CO + *H, with the energy barrier of 0.18 eV, 0.29 eV, 0.72 eV and 0.23 eV for each step, respectively (Fig. 2c, red ring). Therefore, the overflow of OH* produced in the H-assistant *CO_2_ conversion can actually accelerate the CH_4_ dehydrogenation on the Ni_4_–CaO(100) surface, which provides another strong evidence of the synergistic promotions. It is also worth mentioning that the novel pathway of the CH_4_ dehydrogenation disclosed here suggests the important role of the suitable interface of Ni–CaO DFM as it may stride across the carbon deposition in the conventional DRM.

**Fig. 3:**
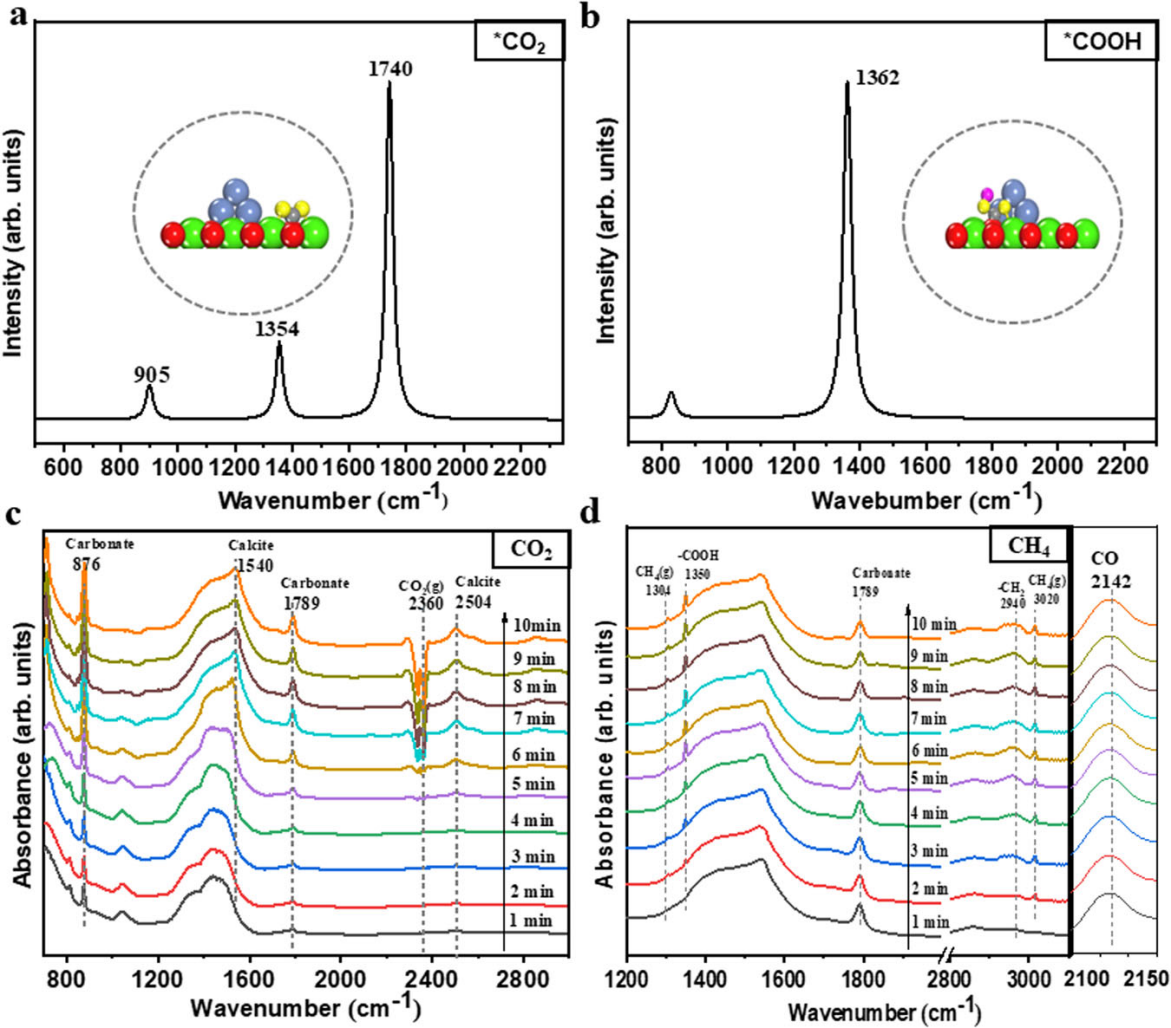
Characteristic spectra of CaL@DRM iCCC processes on the Ni–CaO. The DFPT calculated peaks from the asymmetric stretching of the adsorbed **a** CO_2_ (*CO_2_) and **b** COOH group (*COOH). In situ DRIFTS spectra during **c** the CO_2_ adsorption stage in the atmosphere of 10 vol% CO_2_ balanced with N_2_, and **d** the in situ CO_2_ conversion stage in the atmosphere of 5 vol% CH_4_ balanced with N_2_ of the CaL@DRM iCCC process on the Ni–CaO surface.

We further verified this synergistic mechanism for the CaL@DRM processes through the in-situ diffuse reflectance infrared Fourier transform spectroscopy (DRIFTS) and the density-functional perturbation theory (DFPT) calculation^44–46^. In the CO_2_ capture stage, the optimized carbonate-like adsorbed *CO_2_ species one the CaO(100) surface obtained from DFT calculations was used, and the calculated asymmetric stretching frequencies are at 1740 and 905 cm^−^^1^ (Fig. 3a). Correspondingly, the increasing peaks of the carbonate (*CO_2_) on the CaO surface at 1789 and 876 cm^−1^, and of the calcite (CaCO_3_) at 1540 and 2504 cm^−1^ can be clearly observed in the DRIFTS spectra (Fig. 3c)^47^. After the N_2_ elution, the gas was switched into CH_4_ for the following CO_2_ conversion stage. According to the DFPT calculation, the asymmetric stretching of the adsorbed monodentate *COOH on the CaO(100) gives the peak at 1362 cm^−^^1^ (Fig. 3b). Accordingly, a newly formed peak at 1350 cm^−1^ in the DRIFTS spectra can be also seen and verified to be the key intermediate of *COOH (Fig. 3d)^48^. These results indeed further confirmed the proposed *H-assisted pathway for the *CO_2_ conversion. Moreover, the appearance of a symmetric vibration peak of ν(CH_2_) at 2940 cm^−1^ may also support the *OH-assistant CH_4_ dehydrogenation^48^. As expected, the CO peak at 2142 cm^−1^ gradually increases with the decrease of carbonate peak at 1789 cm^−1^ ^49–51^. Again, since no CO_2_ peak was observed, it can be further clarified that the captured *CO_2_ (CaCO_3_) is indeed directly reduced into CO through the in situ CaL@DRM pathway.

### Control synthesis of Ni–CaO DFMs

**Fig. 4:**
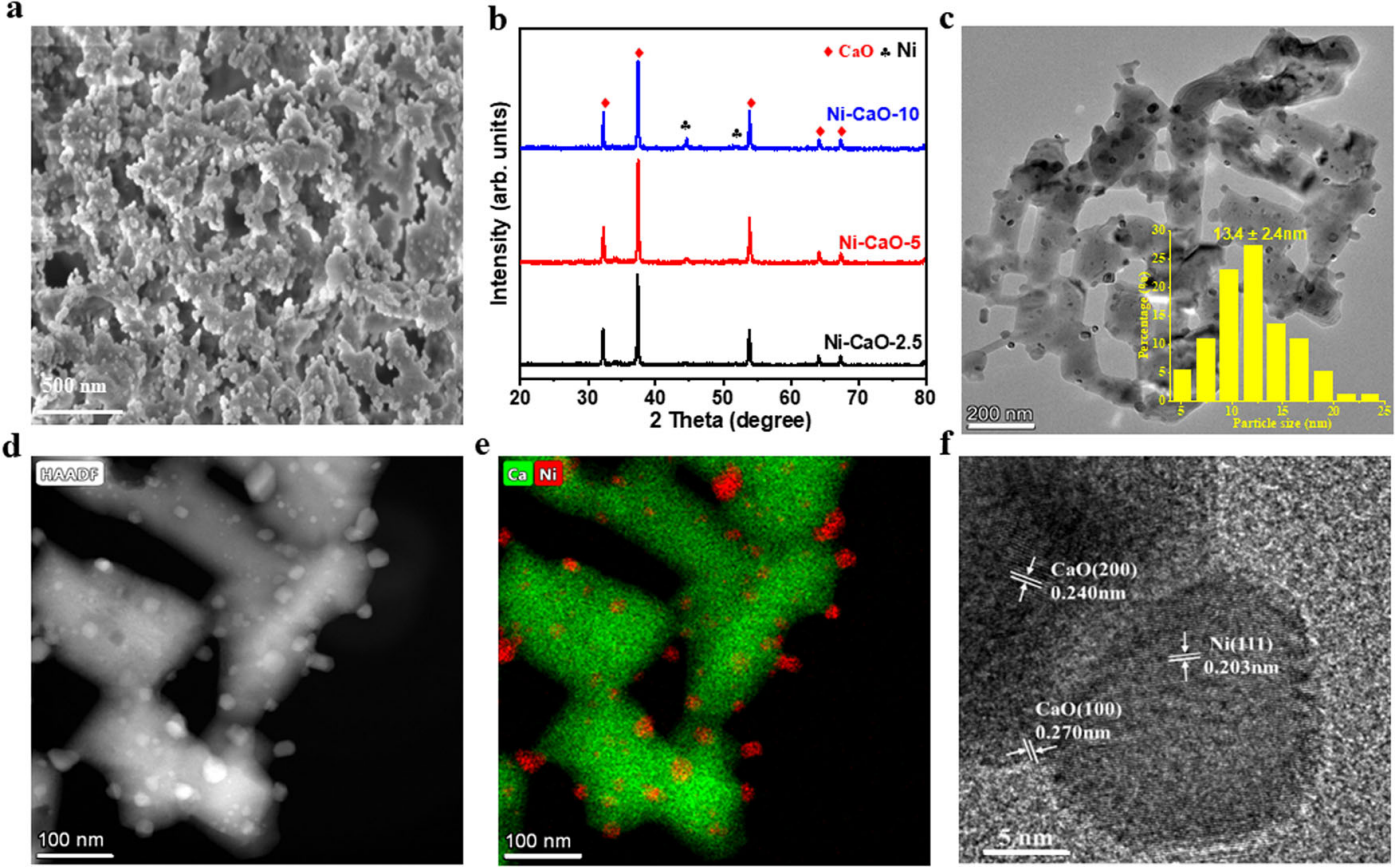
Structural characterizations of Ni–CaO DFMs. **a** Micrometer-scale morphology of Ni–CaO-5 (scale bar = 500 nm). **b** XRD patterns of Ni–CaO-2.5, Ni–CaO-5, Ni–CaO-10. **c** TEM image (scale bar = 200 nm) and size distribution of Ni nanoparticles at Ni–CaO-5. **d** STEM image of Ni–CaO-5 (scale bar = 100 nm). **e** Energy-dispersive X-ray elemental mapping images of Ni–CaO-5. **f** High-resolution TEM of Ni–CaO-5 (scale bar = 5 nm).

From the above results and discussions, it can be expected that the fabrication of proper adsorptive/catalytic interface is essential for the synergistic promotion between CO_2_ capture and in-situ conversion, where the reaction efficiency of the *H-assistant *CO_2_ conversion and the *OH-assistant CH_4_ dehydrogenation should be compatible with each other. In the current work, the Ni–CaO DFMs synthesized by a simple sol–gel method all exhibited a branched coral-like porous morphology (Fig. 4a and Supplementary Fig. 11, Supplementary Table 1). After reduction in H_2_ atmosphere at 700 °C, the X-ray diffraction (XRD) patterns of all samples showed the successful transformation of NiO (JCPD 47-1049) to Ni (JCPD 04-0850), with the dominant CaO crystals being unchanged (JCPD 48-1467) (Fig. 4b and Supplementary Fig. 12). The most of Ni species in Ni–CaO-x are well-distributed throughout the CaO matrix (Supplementary Fig. 13). Along with the increased density of Ni nanoparticles, the average sizes of Ni nanoparticles on the Ni–CaO-2.5, Ni–CaO-5, and Ni–CaO-10 catalysts varied as 13.2 ± 2.5 nm, 13.4 ± 2.4 nm, and 27.6 ± 3.0 nm, respectively (Fig. 4c and Supplementary Fig. 14), suggesting there is a trade-off between the Ni loading density and the size of Ni nanoparticles to achieve the expected adsorptive/catalytic interface in DFM. We estimated the proportion of the adsorptive/catalytic interface by making a distinction between the bonded Ni toward CaO and the free one. On the XPS spectrum of Ni 2*p*_3/2_ of Ni–CaO-x, two peaks of the bonded Ni at 856 eV and the free one at about 854 eV were observed^31^, giving that the bonded Ni of 67, 73, and 56% (atomic concentration) of total Ni on Ni–CaO-2.5, Ni–CaO-5, and Ni–CaO-10, respectively (Supplementary Fig. 15). Therefore, Ni–CaO-5 exhibited the largest adsorptive/catalytic interface as the sizes of the supported Ni nanoparticles kept quite small while the amount of Ni loading was large enough. The scanning transmission electron microscopy (STEM) images complemented by energy-dispersive X-ray elemental mapping (EDX) further demonstrated the homogeneous mixing of catalytic Ni sites with adsorptive CaO sites on the nanoscale (Fig. 4d, e). More specifically, the interface between the closely packed Ni nanoparticle and the CaO support was investigated by the high-resolution TEM (HRTEM) image (Fig. 4f), where the lattice spacings of 0.203 nm of Ni(111) and 0.270 nm of CaO(100) can be clearly seen^52^.

### ICCC performance of Ni–CaO DFMs

**Fig. 5:**
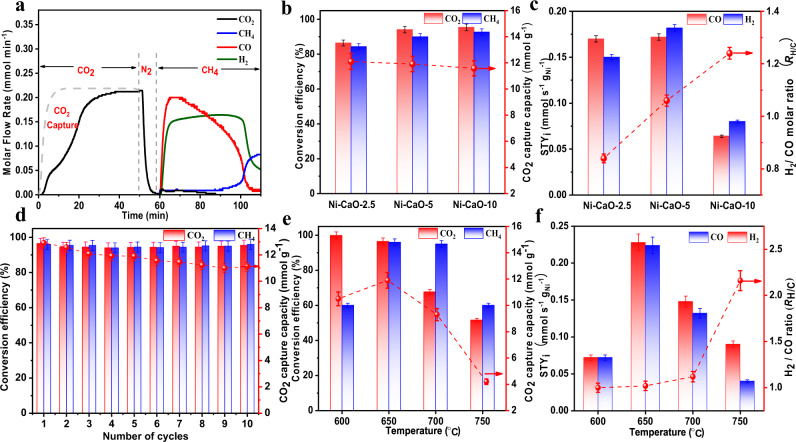
ICCC performance of Ni–CaO DFMs. **a** Molar flow rate of the effluent gas in one cycle of CaL@DRM on the Ni–CaO-5. The effects of Ni loading content on **b** average conversion of CO_2_ and CH_4_ along with CO_2_ capture capacity, and **c** specific yield of H_2_ and CO (average yield per gram of the loaded Ni) along with the *R*_H/C_ in the syngas product at 650 °C. **d** Stability of 10 cyclic CaL@DRM iCCC processes in one Ni–CaO-5(8.2) packed reactor at 650 °C. The effects of temperature on **e** the average conversion of CO_2_ and CH_4_ along with the CO_2_ capture capacity and **f** specific yield of H_2_ and CO (average yield per gram of the loaded Ni) along with the *R*_H/C_ in the syngas product on Ni–CaO-5(8.2). Error bars mean ± standard deviations calculated from three independent measurements.

Then, the iCCC performance was systematically measured on the obtained Ni–CaO-x DFMs in one fixed-bed reactor through consecutive CaL@DRM processes (Fig. 5a and Supplementary Fig. 16). At the fixed temperature of 650 °C, in the stage of CO_2_ capture from the simulated flue gas (10 vol.% CO_2_ balanced with N_2_), all the Ni–CaO-*x* (*x* = 2.5, 5, 10) DFMs exhibited excellent CO_2_ adsorption capacities (Supplementary Table 2), giving slightly decreased values of 12.10 mmol g^−1^, 11.91 mmol g^−1^, and 11.58 mmol g^−1^ along with the increase of Ni loading (Fig. 5b). After N_2_ purge and in the stage of in-situ conversion by switching the gas to 5 vol% CH_4_ balanced with N_2_, the conversions of CH_4_ (CO_2_) reached 86.4% (84.3%), 94.1% (90%), and 95.4% (92.7%) on these catalysts, respectively; and the determined *R*_H/C_ in the syngas products were 0.88, 1.06, and 1.24 (Fig. 5c and Supplementary Table 3). We can clearly see that without sufficient Ni–CaO interfaces on the Ni–CaO-2.5, CH_4_ dehydrogenation can hardly provide enough *H to assistant the *CO_2_ conversion. Instead, the produced H_2_ may participate the reduction of the excessive CO_2_ through the reversed water gas shift reaction (RWGS), resulting in the *R*_H/C_ lower than 1. By contrast, too high loading density of Ni on the Ni–CaO-10 will also lead to the mismatching between the CO_2_ conversion and *OH-assistant CH_4_ dehydrogenation, resulting in the CH_4_ dehydrogenation by itself on the Ni particles, and hence the *R*_H/C_ larger than 1. It was further confirmed the deposited carbon formed on Ni–CaO-10 rather than Ni–CaO-5 in CaL@DRM process by Raman spectra (Supplementary Fig. 17). This can just explain why we observed in the above CH_4_–TPSR measurements that the carbon deposition occurred when rich Ni was loaded on Ni–CaO-10. Accordingly, to improve the comparability in activity among the Ni–CaO DFMs with different Ni loadings, the measured activities of Ni–CaO-*x* were also calculated in terms of per gram of Ni loading, and the Ni–CaO-5 still gave the best performance, with the highest H_2_ and CO yields of 0.182 mmol s^−1^ g_Ni_^−1^ and 0.172 mmol s^−1^ g_Ni_^−1^, respectively (Fig. 5c). It is worth mentioning that such *R*_H/C_ close to unity obtained under so high conversions suggests the complete DRM, with negligible side reactions such as methane decomposition or RWGS. In contrast, for the conventional DRM process with co-feeding of CO_2_ and CH_4_ under the similar operating conditions, Ni–CaO-5 shows much worse activity that the conversions of CO_2_ and CH_4_ significantly decreased to 75.0% and 60.0% at 650 °C, respectively, and the *R*_H/C_ is only about 0.5 (Supplementary Fig. 18), confirming the synergistic promotions between CO_2_ capture and in situ conversion through the CaL@DRM iCCC processes.

To elaborate upon the importance of interface structures for the iCCC performance, we regulate the size of Ni nanoparticles by varying the amount of soft template at the fixed loading of Ni (5 wt%) in the sol-gel process. As the result of the different nanoconfined spaces constructed by the template, a series of Ni–CaO-5(*d*) DFMs with the Ni particle size (*d*) ranging from 8.2 to 17.2 nm were obtained (Supplementary Fig. 19). As listed in Supplementary Table 4, the dispersion degree of Ni increased with the decrease of Ni nanoparticle size, and hence, the smaller Ni nanoparticles, the larger the interface can occur at Ni/CaO. The Ni–CaO-5(*d*) DFMs also gave improved CO_2_ adsorption and conversion with the decreased size of Ni nanoparticles. Specifically, Ni–CaO-5(8.2), with the smallest Ni particle size, exhibited the maximum CO_2_ capture capacity of 12.8 mmol g^-1^ and the best CO_2_ and CH_4_ conversion of 96.5% and 96.0%, respectively, together with the syngas production yield as high as 0.452 mmol s^−1^ g_Ni_^−1^ (Supplementary Fig. 20). Such changing pattern of the iCCC performance with the Ni nanoparticle size provided further evidence that the enlarged Ni/CaO interface can facilitate the synergistic reaction pathways to promote the CaL@DRM reaction on Ni–CaO DFM. Compared with catalysts reported in other studies (Supplementary Table 5), the Ni–CaO-5(8.2) DFM produced by the modified sol–gel method showed the highest CO_2_ conversion and CH_4_ conversion of 96.5% and 96.0%, respectively. More significantly, the H_2_/CO molar ratio in the elution profile was nearly 1, suggesting that Ni–CaO-5(8.2) DFM had a much better resistance to carbon deposition compared with the Ni-Ca@Zr and Ni–CaO catal@sorbent^26,53^.

In addition, to realize the consecutive reactions of the exothermic CO_2_ capture and the endothermic conversion in the same reactor for the iCCC processes, the reaction temperature plays a pivotal role^6,54^. The syngas production is determined not only by the catalytic efficiency in the in situ conversion, but also the CO_2_ capacity in the CO_2_ capture stage. In the temperature range of 600–750 °C, in consistence with the CO_2_–TPSR curve, the CO_2_ adsorption capacity of Ni–CaO-5(8.2) shows a maximum at 650 °C (Fig. 5e); while the CO_2_ conversion efficiency decreases with increasing temperature and the CH_4_ conversion efficiency approaches the maximum at 700 °C, and overall, the production of H_2_ and CO shows an outstanding performance at 650 °C (Fig. 5 f). Notably, the *R*_H/C_ booms up to 2.4 at 750 °C, suggesting the excessive temperature will lead to the more significant CH_4_ dehydrogenation, which may even cause the coking on the Ni-based catalysts^55^. Accordingly, 650 °C is the optimized temperature for the consecutive CaL@DRM processes. In this work, 10 successive runs at 650 °C in the Ni–CaO-5(8.2) packed reactor were conducted, and the results showed an extremely stable CO_2_ and CH_4_ conversions of 96.5% and 96.0%, respectively, with only a slight decrease in the cyclic CO_2_ capture capacity (Fig. 5d). Moreover, there was no obvious carbon deposition on Ni–CaO-5(8.2) after the 10 cyclic runs (Supplementary Fig. 21), demonstrating the excellent performance of this synergistic CaL@DRM iCCC processes.

## Discussion

In summary, we demonstrated the synergistic effects between CO_2_ capture and in-situ conversion through the consecutive CaL@DRM processes on the Ni–CaO DFM catalysts. The rich Ni–CaO interfaces with the catalytic sites and adsorptive sites intimately closed with each other promote the CH_4_ dehydrogenation on the catalytic Ni site to form *H intermediate which can overflow to the nearby CaO surface and react with the captured *CO_2_ there to facilitate its reduction; at the same time, the *OH intermediate produced from the *CO_2_ conversion can reversely participate in the CH_4_ dehydrogenation and induce the *CH_2_ oxidation to form CO. The critical step for the formation of *COOH intermediate from CO_2_ during the CaL@DRM processes was captured by the in-situ DRIFTS spectra, demonstrating the reliability of the proposed synergistic promotion mechanism. Furthermore, the adsorptive/catalytic interface on Ni–CaO was maximized by balancing the loading density of Ni and its aggregation growth on porous CaO. After optimizing the temperature matching between the exothermic CO_2_ capture and endothermic conversion in one fixed-bed reactor, the extraordinary synergistic promotions of CaL@DRM performance were achieved at 650 °C that the CO_2_ and CH_4_ conversions were as high as 96.5% and 96.0%, respectively, with the syngas production yield of 0.452 mmol s^−^^1^ g_Ni_^−1^. Due to the synergistic effects on facilitating the pathways of CaCO_3_ regeneration and CH_4_ dehydrogenation, the bottleneck deactivation problems of the CaO sintering and carbon deposition were successfully surpassed, resulting in high recycle-stability. We anticipate that our findings of the synergistic promotions in coupling carbon capture and in-situ conversion could guide the design of highly efficient DFMs for iCCC processes.

## Methods

### Chemicals

Calcium nitrate tetrahydrate (Ca(NO_3_)_2_·4H_2_O, 99.99%), nickel nitrate hexahydrate (Ni(NO_3_)_2_·6H_2_O, 99.99%), urea(CO(NH_2_)_2_, 99.99%) and citric acid monohydrate (C_6_H_10_O_8_, 99.99%) were purchased from Sinopharm. All chemicals were in the analytical reagent-grade and used as received. Deionized water was used in all synthesis and washing processes.

### Synthesis of Ni–CaO-*x* dual function materials (DFMs)

Ni–CaO-*x* DFMs were prepared by a simple one-pot sol–gel approach and a subsequent calcination. Typically, 8.43 g of Ca(NO_3_)_2_·4H_2_O and 7.50 g of citric acid monohydrate were dissolved in 20 mL of distilled water at room temperature under stirring for 0.5 h. Then specific amounts of Ni(NO_3_)_2_·6H_2_O were added into the mixture under stirring for an additional 0.5 h. Then, the mixture was continuously stirred at 90 °C to form a translucent pale-green sol. The sol was transferred in an oven at 120 °C for 12 h to obtain a dried gel. The dried gel was calcined in a muffle furnace under air at 800 °C for 4 h at a temperature ramp rate of 2 °C min^−1^ to produce the Ni–CaO-*x* dual function materials, where *x* represents the weight fraction of Ni. The DFMs were fully reduced by H_2_ at 700 °C for 2 hours before use. In addition, pure CaO was also prepared as a reference sample according to the above-mentioned procedures.

For Ni–CaO-5(*d*) DFMs loading the same Ni loading (5 wt%), prepared by the modified sol–gel method, different molar ratio (0.25, 0.5, 1, and 2) of soft organic template (citric acid and urea) with metal precursor were dissolved in water. The other prepared steps are the same as above. But the dried gel firstly was calcined under N_2_ at 800 °C for 2 h and then calcined under air at 600 °C for 2 h. Ni–CaO-5(*d*) samples under investigation have varying Ni mean particle sizes (*d*), as listed in Supplementary Table 4.

### Characterization

Powder X-ray diffraction (PXRD) patterns were obtained on a Bruker D8 Advance X-ray powder diffractometer equipped with a Cu sealed tube (λ = 1.54 Å) at a scan rate of 0.02 s^−1^. Scanning electron microscopy (SEM) was conducted on a Helios G4 UC SEM-FIB (15 kV). Samples were pre-treated via Pt sputtering before the observation. Transmission electron microscopy (TEM) and Scanning transmission electron microscopy (STEM) images were performed on a Thermo-Fisher Talos F200X (FETEM, 200 kV) equipped with an energy dispersive spectrometer (EDS) for determining the elemental distribution. High angle annular dark field (HAADF)-STEM images were recorded by using a convergence semi angle of 11 mrad, and inner and outer collection angles of 59 and 200 mrad, respectively. Nitrogen adsorption-desorption isotherms were measured at 77 K using a Micromeritics ASAP-2020, from which the specific surface area and the pore size distribution of samples were calculated based on the Brunauer–Emmett–Teller (BET) model and the Barrett–Joyner–Halenda (BJH) approach, respectively. Before the measurement, the samples were degassed at 200 °C for 5 h. Elemental analysis was performed using an inductively coupled plasma atomic emission spectrometer (ICP, Varia 710 ES). X-ray photoelectron spectroscopy (XPS) of the samples were recorded on a Thermo-Scientific K-Alpha Spectrometer. All peaks were corrected by setting the C 1 *s* peak of 284.6 eV as the reference. Fourier transform infrared spectra (FTIR) was performed on a NEXUS 470 spectrometer in the range of 4000–400 cm^−1^. Prior to the measurement, the sample was mixed with potassium bromide and pressed into a wafer.

The temperature-programmed reduction in hydrogen (H_2_-TPR) was carried out on an automated chemisorption flow analyzer (Autochem 2720, Micromeritics), equipped with a thermal conductivity detector to optimize the reduction conditions of DFMs. In a typical experiment, 50 mg of the calcined sample was loaded in a quartz reactor and heated to 300 °C in the nitrogen atmosphere (50 mL min^−1^) for the dehydration. After the temperature was cooled down to 50 °C, the gas was switched into 10 vol% H_2_ balanced with Ar (50 mL min^−1^). Subsequently, the temperature was increased again with a heating rate of 10 °C min^−1^.

The temperature programmed surface reaction (TPSR) was performed on Autochem 2720. The performance of the Calcium looping (CaL) CO_2_ capture process, the CH_4_ dehydrogenation, and the integrated CO_2_ capture and conversion of CaL@DRM processes was investigated by the CO_2_-TPSR, CH_4_-TPSR, and CH_4_-TPSR with CO_2_ pre-adsorption, respectively. Prior to each test, the sample (0.1 g) was pretreated under 10 vol% H_2_ balanced with N_2_ at 700 °C for 30 min. For the CaL process determined by the CO_2_-TPSR test, samples were heated from 50 to 900 °C with a heating rate of 10 °C min^−^^1^ under the gas flow of 10 vol% CO_2_ balanced with N_2_ at a rate of 50 ml min^−^^1^. For the CH_4_ dehydrogenation process determined by the CH_4_-TPSR test, the operation conditions are the same except the gas flow changed into 5 vol% CH_4_ balanced with N_2_. For the CaL@DRM processes, the sample was exposed to a gas flow of 10 vol.% CO_2_ balanced with N_2_ at a rate of 50 ml min^−1^ for 30 min at 650 °C to make DFMs saturated with CO_2_. Then the sample was cooled down to 50 °C. Afterwards, the CH_4_-TPSR test was carried out under the same conditions as above. The nondispersive infrared analyzer (Smart Pro, Shandon) was used to monitor the concentration changes of each substance (CO, CO_2_, CH_4_, and H_2_) participated during all TPSRs in effluents.

The produced intermediates during CaL@DRM process were monitored by an in situ diffuse reflectance infrared Fourier transform spectroscopy (DRIFTS, Thermo-Scientific, Nicolet 6700), equipped with a diffuse reflection attachment reaction cell (DRK-3 Praying Mantis Harrik). The spectra were recorded in the range of 3000–750 cm^−1^ per 64 scans with a resolution of 4 cm^−1^. Prior to each testing, the sample (∼5 mg) was pre-reduced in the atmosphere of 10 vol% H_2_ balanced with He (25 ml min^−1^) at 700 °C for 2 h. Then, the gas was switched into N_2_ to remove the residual H_2_ for 30 min by decreasing the temperature to 600 °C, and the background reference signals were collected. The CO_2_ capture was performed in the atmosphere of 10 vol% CO_2_ balanced with N_2_ (50 mL min^−1^) for 10 min. After the N_2_ elution for 8 min, the in situ CO_2_ conversion was performed by switching the gas into 5 vol% CH_4_ balanced with N_2_ (50 mL min^−1^) for another 10 min.

### CO_2_ capture and in situ conversion test

The CO_2_ capture and in situ conversion (CaL@DRM processes) were conducted in one fixed-bed column. The flow rates of N_2_, CO_2_, H_2_, and CH_4_ were controlled by the mass flow controllers (Horiba Metron), respectively. The products in the outlet gas were analyzed by the gas chromatography (GC, Haixin 950) with a thermal conductivity detector (TCD) and a flame ionization detector (FID). In addition, an in situ nondispersive infrared analyzer (Smart Pro, Shandon) was used to monitor the concentration changes of CO, CH_4_, H_2_, and CO_2_ continuously. In a typical experiment, approximately 0.1 g DFMs was added to a quartz tube (Φ10 mm × 150 mm) and packed with height of about 10 mm, then placed in the reactor furnace. The first step was the sample prereduction, which was carried out at 700 °C in the gas flow of 10 vol% H_2_ balanced with N_2_ at a rate of 50 ml min^−1^ for 2 h. The second step was the CO_2_ capture, in which the gas was switched to the simulated flue gas of 10 vol% CO_2_ balanced with N_2_ at 50 ml min^−1^ (WHSV = 30 L g^−1^ h^−^^1^) and at a specific temperature such as 600, 650, 700, and 750 °C for 1 h. The third step was the purge. The pipeline was purged with pure N_2_ for 5 min. The fourth step was the in situ conversion, in which the temperature was kept the same as in CO_2_ capture, and the gas was switched to 5 vol% CH_4_ balanced with N_2_ at a flowrate of 50 ml min^−1^. Moreover, we performed the blank test in the same fixed bed under the same operating conditions by using an inert material (SiO_2_) with similar particle size to that of DFMs.

The CO_2_ adsorption capacity (*q)* was calculated by Eq. (1)

1 $$q=\frac{{\int }_{0}^{{{{{{\rm{ts}}}}}}}[{F}_{{{{{{{\rm{CO}}}}}}}_{2},{{{{{\rm{in}}}}}}}-{F}_{{{{{{{\rm{CO}}}}}}}_{2},{{{{{\rm{out}}}}}}}({{{{{\rm{t}}}}}})]dt}{{M}_{0}}$$

where ${F}_{{{{{{\rm{CO}}}}}}_{2},{{{{{\rm{in}}}}}}}$ is the CO_2_ molar flow rate in the inlet gas of the simulated flue gas, ${F}_{{{{{{\rm{CO}}}}}}_{2},{{{{{\rm{out}}}}}}}$ is the CO_2_ molar flow rate in the outlet gas, *t*s is the duration time of the capture step, and *M*_0_ is the sample mass.

The CO_2_ and CH_4_ conversion efficiency was calculated by Eq. (2)

2 $${{{{{{\rm{X}}}}}}}_{i}=\frac{{\int }_{0}^{{{{{{\rm{ts}}}}}}}[{F}_{{{{{{\rm{i}}}}}},{{{{{\rm{in}}}}}}}-{F}_{{{{{{\rm{i}}}}}},{{{{{\rm{out}}}}}}}({{{{{\rm{t}}}}}})]dt}{{{{{{{\rm{F}}}}}}}_{{{{{{\rm{i}}}}}},{{{{{\rm{in}}}}}}}\times ts}\times 100\%$$

where X_i_ (%) is the conversion of CO_2_ or CH_4_, *F*_i,in_, and *F*_i,out_, represent the molar flow rate of CO_2_ or CH_4_ in the inlet gas and the outlet gas, respectively. *t*s is the duration time of the in-situ conversion step.

The average space time yield (STY_i_) of CO or H_2_ was calculated by Eq. (3):

3 $${{{{{{\rm{STY}}}}}}}_{i}=\frac{{\int }_{0}^{{{{{{\rm{ts}}}}}}}[{F}_{{{{{{\rm{i}}}}}},{{{{{\rm{out}}}}}}}({{{{{\rm{t}}}}}})]dt}{{M}_{0}\times ts}$$

where *F*_i,out_ represents the molar flow rate of CO or H_2_ in the outlet gas, *t*s is the duration time of the in-situ conversion step, and *M*_0_ is the sample mass.

The H_2_/CO molar ratio (*R*_H/C_) of syngas was determined by the ratio between H_2_ and CO concentrations in the outlet gas.

For comparison, the conventional DRM test was also conducted as above, except that the CO_2_ capture step was skipped, and the feeding gas was changed into the mixture of 10 vol% CO_2_ and 10 vol% CH_4_ balanced with N_2_.

### DFT calculations

All spin-polarized DFT calculations were carried out using the Vienna Ab–Initio Simulation Package (VASP)^56,57^. The projector augmented wave (PAW) method^58^ and the Perdew–Burke–Ernzerhof (PBE)^59^ functional under the generalized gradient approximation (GGA)^60^ were applied throughout the calculations. The kinetic energy cut-off was set to 400 eV, and the force threshold in structure optimization was 0.05 eV Å^−1^. We used a large vacuum gap of 15 Å to eliminate the interactions between neighboring slabs. By adopting these calculation settings, the optimized lattice constant of CaO is 4.80 Å, which is in good agreement with the experimental value of 4.80 Å^61^. For the model construction, the Ni_4_ cluster to simplify the theoretical calculation model cluster is widely used as a classic which could well reflect the characteristic of simulated nanoparticles in the DFT calculation^62–64^. Therefore, we built a p (4×4) surface slab containing five atom layers for CaO(100) and Ni_4_ supported CaO(100) surfaces. The top four atom layers of these slabs were allowed to fully relax, while the bottom atom layer was kept fixed to mimic the bulk region. A 2 × 2 × 1 k-point mesh was used in calculations of all these models. Calculation of the IR spectrum for the adsorbed of CO_2_ and COOH species on the Ni_4_–CaO(100) surfaces using the Born charges and the density functional perturbation theory (DFPT)^45,46^. For comparison, we also constructed the model Ni–CaO DFM with Ni 13-atom Ni clusters being supported on the CaO(100) substrate, namely Ni_13_–CaO(100).

The transition states (TS) of surface reactions were located using a constrained optimization scheme and were verified when (i) all forces on the relaxed atoms vanish and (ii) the total energy is a maximum along the reaction coordination, but it is a minimum with respect to the rest of the degrees of freedom^65–67^. The adsorption energy of species *X* on the surface (*E*_ads_(*X*)) was calculated by the Eq. (4):

4 $${E}_{{{{{{\rm{ads}}}}}}}(X)=-({E}_{X/{{{{\rm{slab}}}}}}-{E}_{{{{{\rm{slab}}}}}}-{E}_{X})$$

where *E*_*X*/slab_ is the calculated total energy of the adsorption system, while *E*_*X*/slab_ and *E*_*X*_ are calculated energies of the clean surface and the gas phase molecule *X*, respectively. Obviously, a positive value of *E*_ads_(*X*) indicates an exothermic adsorption process, and the more positive the *E*_ads_(*X*) is, the more strongly the adsorbate *X* binds to the surface.

## Supplementary information

Supplementary Information

Peer Review File

## Data Availability

The data that support the findings of this study are available within the article and Supplementary Information or from the corresponding authors on reasonable request.

## References

[1] Allen, M. et al. Technical summary: global warming of 1.5 °C. An IPCC Special Report on the impacts of global warming of 1.5 °C above pre-industrial levels and related global greenhouse gas emission pathways, in the context of strengthening the global response to the threat of climate change, sustainable development, and efforts to eradicate poverty. Intergovernmental Panel on Climate Change (2019).

[2] Gao W (2020). Industrial carbon dioxide capture and utilization: state of the art and future challenges. Chem. Soc. Rev..

[3] Sullivan I (2021). Coupling electrochemical CO_2_ conversion with CO_2_ capture. Nat. Catal..

[4] Cheng D (2022). Catalytic synthesis of formamides by integrating CO_2_ capture and morpholine formylation on supported Iridium catalyst. Angew. Chem. Int. Ed..

[5] Sen R, Goeppert A, Kar S, Prakash GKS (2020). Hydroxide based integrated CO_2_ capture from air and conversion to methanol. J. Am. Chem. Soc..

[6] Shao B (2021). CO_2_ capture and in-situ conversion: recent progresses and perspectives. Green. Chem. Eng..

[7] Li M, Irtem E, Iglesias van Montfort H-P, Abdinejad M, Burdyny T (2022). Energy comparison of sequential and integrated CO_2_ capture and electrochemical conversion. Nat. Commun..

[8] Li L (2022). Continuous CO_2_ capture and selective hydrogenation to CO over Na-promoted Pt nanoparticles on Al_2_O_3_. ACS Catal..

[9] Shao B (2021). Heterojunction-redox catalysts of Fe_x_Co_y_Mg_10_CaO for high-temperature CO_2_ capture and in situ conversion in the context of green manufacturing. Energy Environ. Sci..

[10] Jo S (2022). Perspective on sorption enhanced bifunctional catalysts to produce hydrocarbons. ACS Catal..

[11] Al-Mamoori A, Lawson S, Rownaghi AA, Rezaei F (2020). Oxidative dehydrogenation of ethane to ethylene in an integrated CO_2_ capture-utilization process. Appl. Catal. B Environ..

[12] Lawson S (2022). Adsorption-enhanced bifunctional catalysts for in situ CO_2_ capture and utilization in propylene production: a proof-of-concept study. ACS Catal.

[13] Hanak DP, Anthony EJ, Manovic V (2015). A review of developments in pilot-plant testing and modelling of calcium looping process for CO_2_ capture from power generation systems. Energy Environ. Sci..

[14] Naeem MA (2018). Optimization of the structural characteristics of CaO and its effective stabilization yield high-capacity CO_2_ sorbents. Nat. Commun..

[15] Tang Y (2019). Rh single atoms on TiO_2_ dynamically respond to reaction conditions by adapting their site. Nat. Commun..

[16] Duyar MS, Treviño MAA, Farrauto RJ (2015). Dual function materials for CO_2_ capture and conversion using renewable H_2_. Appl. Catal. B Environ..

[17] Sun H (2019). Dual functional catalytic materials of Ni over Ce-modified CaO sorbents for integrated CO_2_ capture and conversion. Appl. Catal. B Environ..

[18] Zhao Y, Jin B, Liang Z (2019). Synergistic enhanced Ca–Fe chemical looping reforming process for integrated CO_2_ capture and conversion. Ind. Eng. Chem. Res..

[19] Zhu Q (2022). Enhanced CO_2_ utilization in dry reforming of methane achieved through nickel-mediated hydrogen spillover in zeolite crystals. Nat. Catal..

[20] Dunstan MT, Donat F, Bork AH, Grey CP, Muller CR (2021). CO_2_ capture at medium to high temperature using solid oxide-based sorbents: fundamental aspects, mechanistic insights, and recent advances. Chem. Rev..

[21] Bermejo-López A, Pereda-Ayo B, González-Marcos JA, González-Velasco JR (2019). Mechanism of the CO_2_ storage and in situ hydrogenation to CH_4_. Temperature and adsorbent loading effects over Ru-CaO/Al_2_O_3_ and Ru-Na_2_CO_3_/Al_2_O_3_ catalysts. Appl. Catal. B Environ..

[22] Bermejo-López A, Pereda-Ayo B, González-Marcos JA, González-Velasco JR (2019). Ni loading effects on dual function materials for capture and in-situ conversion of CO_2_ to CH_4_ using CaO or Na_2_CO_3_. J. CO2 Util..

[23] Bermejo-López A, Pereda-Ayo B, Onrubia-Calvo JA, González-Marcos JA, González-Velasco JR (2022). Tuning basicity of dual function materials widens operation temperature window for efficient CO_2_ adsorption and hydrogenation to CH_4_. J. CO2 Util..

[24] Yoshida N, Hattori T, Komai E, Wada T (1999). Methane formation by metal-catalyzed hydrogenation of solid calcium carbonate. Catal. Lett..

[25] Jo SB (2020). A novel integrated CO_2_ capture and direct methanation process using Ni/CaO catal-sorbents. Sustain. Energy Fuels.

[26] Jo SB (2020). CO_2_ green technologies in CO_2_ capture and direct utilization processes: methanation, reverse water-gas shift, and dry reforming of methane. Sustain. Energy Fuels.

[27] Reller A, Padeste C, Hug P (1987). Formation of organic carbon compounds from metal carbonates. Nature.

[28] Liu X (2018). Design of efficient bifunctional catalysts for direct conversion of syngas into lower olefins via methanol/dimethyl ether intermediates. Chem. Sci..

[29] Song Y (2020). Dry reforming of methane by stable Ni–Mo nanocatalysts on single-crystalline MgO. Science.

[30] Kurlov A (2020). Exploiting two-dimensional morphology of molybdenum oxycarbide to enable efficient catalytic dry reforming of methane. Nat. Commun..

[31] Tian S, Yan F, Zhang Z, Jiang J (2019). Calcium-looping reforming of methane realizes in situ CO_2_ utilization with improved energy efficiency. Sci. Adv..

[32] Liu P (2022). Synergy between palladium single atoms and nanoparticles via hydrogen spillover for enhancing CO_2_ photoreduction to CH_4_. Adv. Mater..

[33] Zhang X (2021). High-performance binary Mo–Ni catalysts for efficient carbon removal during carbon dioxide reforming of methane. ACS Catal..

[34] Gueddida S, Lebègue S, Pasc A, Dufour A, Badawi M (2021). Ab initio investigation of the adsorption of phenolic compounds, CO, and H_2_O over metallic cluster/silica catalysts for hydrodeoxygenation process. Appl. Surf. Sci..

[35] Gueddida S, Badawi M, Lebègue S (2020). Grafting of iron on amorphous silica surfaces from ab initio calculations. J. Chem. Phys..

[36] Wu P (2019). Cooperation of Ni and CaO at interface for CO_2_ reforming of CH_4_: a combined theoretical and experimental study. ACS Catal..

[37] Wang Y, Li Y, Yang L, Fan X, Chu L (2022). Revealing the effects of Ni on sorption-enhanced water-gas shift reaction of CaO for H_2_ production by density functional theory. Process Saf. Environ. Prot..

[38] Grimme S (2006). Semiempirical GGA-type density functional constructed with a long-range dispersion correction. J. Comput. Chem..

[39] Bučko T, Hafner J, Lebègue S, Ángyán JG (2010). Improved description of the structure of molecular and layered crystals: ab initio DFT calculations with van der Waals corrections. J. Phys. Chem. A.

[40] Gueddida S, Lebègue S, Badawi M (2020). Interaction between transition metals (Co, Ni, and Cu) systems and amorphous silica surfaces: a DFT investigation. Appl. Surf. Sci..

[41] Kattel S, Ramírez PJ, Chen JG, Rodriguez JA, Liu P (2017). Active sites for CO_2_ hydrogenation to methanol on Cu/ZnO catalysts. Science.

[42] Kattel S, Liu P, Chen JG (2017). Tuning selectivity of CO_2_ hydrogenation reactions at the metal/oxide interface. J. Am. Chem. Soc..

[43] Kattel S, Yan B, Yang Y, Chen JG, Liu P (2016). Optimizing binding energies of key intermediates for CO_2_ hydrogenation to methanol over oxide-supported copper. J. Am. Chem. Soc..

[44] Foucaud Y (2021). Adsorption mechanisms of fatty acids on fluorite unraveled by infrared spectroscopy and first-principles calculations. J. Colloid Interface Sci..

[45] Baroni S, de Gironcoli S, Dal Corso A, Giannozzi P (2001). Phonons and related crystal properties from density-functional perturbation theory. Rev. Mod. Phys..

[46] Karhánek D, Bučko T, Hafner J (2010). A density-functional study of the adsorption of methane-thiol on the (111) surfaces of the Ni-group metals: II. Vibrational spectroscopy. J. Phys..

[47] Mutch GA, Anderson JA, Vega-Maza D (2017). Surface and bulk carbonate formation in calcium oxide during CO_2_ capture. Appl. Energy.

[48] Barman S, Singh A, Rahimi FA, Maji TK (2021). Metal-free catalysis: a redox-active donor-acceptor conjugated microporous polymer for selective visible-light-driven CO_2_ reduction to CH_4_. J. Am. Chem. Soc..

[49] Xu S (2019). Sustaining metal–organic frameworks for water–gas shift catalysis by non-thermal plasma. Nat. Catal..

[50] Xu S (2020). CO poisoning of Ru catalysts in CO_2_ hydrogenation under thermal and plasma conditions: a combined kinetic and diffuse reflectance infrared fourier transform spectroscopy–mass spectrometry study. ACS Catal..

[51] Cherevotan A (2021). Operando generated ordered heterogeneous catalyst for the selective conversion of CO_2_ to methanol. ACS Energy Lett..

[52] Guo H (2020). The effect of incorporation Mg ions into the crystal lattice of CaO on the high temperature CO_2_ capture. J. CO2 Util..

[53] Hu J, Hongmanorom P, Galvita VV, Li Z, Kawi S (2021). Bifunctional Ni-Ca based material for integrated CO_2_ capture and conversion via calcium-looping dry reforming. Appl. Catal. B Environ..

[54] Cheng K (2016). Direct and highly selective conversion of synthesis gas into lower olefins: design of a bifunctional catalyst combining methanol synthesis and carbon–carbon coupling. Angew. Chem. Int. Ed..

[55] Chen Q (2017). Temperature-dependent anti-coking behaviors of highly stable Ni-CaO-ZrO_2_ nanocomposite catalysts for CO_2_ reforming of methane. Chem. Eng. J..

[56] Kresse G, Furthmüller J (1996). Efficient iterative schemes for ab initio total-energy calculations using a plane-wave basis set. Phys. Rev. B.

[57] Kresse G, Hafner J (2000). First-principles study of the adsorption of atomic H on Ni (111), (100) and (110). Surf. Sci..

[58] Blöchl PE (1994). Projector augmented-wave method. Phys. Rev. B.

[59] Perdew JP, Burke K, Ernzerhof M (1996). Generalized gradient approximation made simple. Phys. Rev. Lett..

[60] Teter MP, Payne MC, Allan DC (1989). Solution of schrodinger’s equation for large systems. Phys. Rev. B.

[61] Shen CH, Liu RS, Lin JG, Huang CY (2001). Phase stability study of La_1.2_Ca_1.8_Mn_2_O_7_. Mater. Res. Bull..

[62] Zuo Z (2018). Dry reforming of methane on single-site Ni/MgO catalysts: importance of site confinement. ACS Catal..

[63] Liu H (2014). CH_4_ dissociation on the perfect and defective MgO(001) supported Ni_4_. Fuel.

[64] Guo Y, Feng J, Li W (2017). Effect of the Ni size on CH_4_ /CO_2_ reforming over Ni/MgO catalyst: a DFT study. Chin. J. Chem. Eng..

[65] Alavi A, Hu P, Deutsch T, Silvestrelli PL, Hutter J (1998). CO oxidation on Pt(111): an ab initio density functional theory study. Phys. Rev. Lett..

[66] Liu Z-P, Hu P (2003). General rules for predicting where a catalytic reaction should occur on metal surfaces: a density functional theory study of C−H and C−O bond breaking/making on flat, stepped, and kinked metal surfaces. J. Am. Chem. Soc..

[67] Michaelides A (2003). Identification of general linear relationships between activation energies and enthalpy changes for dissociation reactions at surfaces. J. Am. Chem. Soc..

