# Supplementary material for: Synergistic promotions between CO2 capture and in-situ conversion on Ni-CaO composite catalyst
Source: Nat Commun. 2023 Feb 22;14:996. doi: 10.1038/s41467-023-36646-2 (PMC9947161; doi:10.1038/s41467-023-36646-2)
Supplement: Supplementary file 1 — Supplementary Information [file 41467_2023_36646_MOESM1_ESM.pdf]

Supplementary Information for

Synergistic Promotions between CO_2 Capture and in-situ Conversion on Ni-CaO Composite Catalyst

Bin Shao[1,†], Zhi-Qiang Wang[1,†], Xue-Qing Gong[1,*], Honglai Liu[1,2], Feng Qian[3], P. Hu[1,4] & Jun Hu[1,*]

[1] Key Laboratory for Advanced Materials and Joint International Research Laboratory for Precision Chemistry and Molecular Engineering, Feringa Nobel Prize Scientist Joint Research Center, Centre for Computational Chemistry and Research Institute of Industrial Catalysis, School of Chemistry and Molecular Engineering, East China University of Science and Technology, 130 Meilong Road, Shanghai, 200237, China.

[2] State Key Laboratory of Chemical Engineering, School of Chemical Engineering, East China University of Science and Technology, 130 Meilong Road, Shanghai 200237, China

[3] Key Laboratory of Advanced Control and Optimization for Chemical Processes of Ministry of Education, School of Information Science and Engineering, East China University of Science and Technology, 130 Meilong Road, Shanghai 200237, China

[4] School of Chemistry and Chemical Engineering, The Queen's University of Belfast, Belfast BT9 5AG, United Kingdom

† These authors contributed equally: Bin Shao, Zhi-Qiang Wang

Correspondence to: J. H and X.-Q. G

*E-mails: junhu@ecust.edu.cn; xgong@ecust.edu.cn

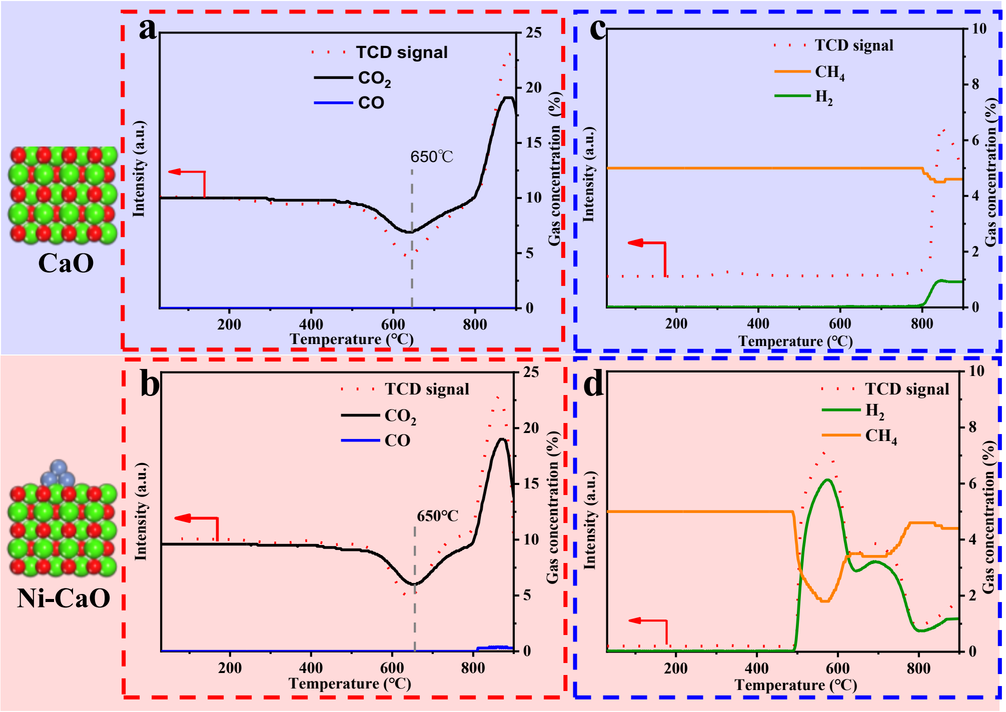

Supplementary Fig. 1 Temperature-programmed surface reaction (TPSR) performance on pure CaO and Ni-CaO-10. a-b CO_2-TPSR; **c-d** CH_4-TPSR.

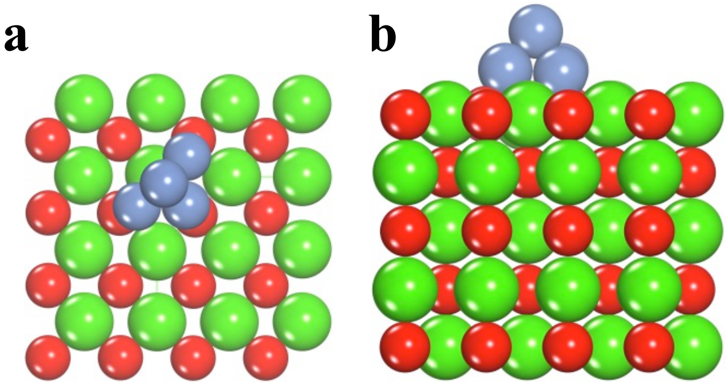

Supplementary Fig. 2 Calculated structures of Ni_4-CaO(100) surface. a side view; **b** top view. Red: O atoms; green: Ca atoms; blue: Ni atoms. This notation is used throughout the paper.

Ni_4-CaO(100) coordinate file (for VASP calculations)

AutoCreatByScript: O Ca Ni
1.00000000000000
13.6061499999999995 0.0000000000000000 0.0000000000000000
0.0000000000000000 13.6061499999999995 0.0000000000000000
0.0000000000000000 0.0000000000000000 24.6209999999999987
O Ca Ni
80 80 4
Selective dynamics
Direct
0.0615870000000029 0.0603520000000017 0.0000000000000000
0.5615870000000029 0.0603520000000017 0.0000000000000000
0.3115870000000029 0.0603520000000017 0.0000000000000000
0.8115870000000029 0.0603520000000017 0.0000000000000000
0.3115870000000029 0.3103520000000017 0.0000000000000000
0.8115870000000029 0.3103520000000017 0.0000000000000000
0.0615870000000029 0.3103520000000017 0.0000000000000000
0.5615870000000029 0.3103520000000017 0.0000000000000000
0.5615870000000029 0.5603520000000017 0.0000000000000000
0.3115870000000029 0.5603520000000017 0.0000000000000000
0.8115870000000029 0.5603520000000017 0.0000000000000000
0.0615870000000029 0.5603520000000017 0.0000000000000000
0.3115870000000029 0.8103520000000017 0.0000000000000000
0.8115870000000029 0.8103520000000017 0.0000000000000000
0.0615870000000029 0.8103520000000017 0.0000000000000000
0.5615870000000029 0.8103520000000017 0.0000000000000000
0.1873600322894034 0.1847476595971917 0.0977575378118760

0.6873360344279256	0.1845970306335858	0.0977353050171388
0.4373103717033970	0.1845967111341119	0.0977818455861648
0.9373478709559172	0.1847742859907677	0.0977502824993045
0.4372595325674458	0.4347183619428091	0.0979467148718033
0.9374930357045977	0.4346743297084689	0.0978029456997183
0.1874047819201812	0.4347352767295179	0.0978616496303582
0.6874832456953112	0.4345967987603468	0.0977315065232426
0.1873829116506056	0.6845411019681473	0.0977908392010816
0.6872956563799120	0.6847460501515448	0.0979506495382918
0.4372710361814341	0.6846692332774572	0.0978641623016034
0.9373416487627871	0.6846521151491922	0.0977911926194159
0.4374653125757796	0.9346174864683301	0.0979011825829009
0.9373675704734739	0.9346741629768518	0.0978148959508097
0.1871316512943403	0.9349070786545370	0.0976827548873615
0.6873048980490770	0.9346550948192228	0.0979342456015598
0.0629774918726319	0.0590682325579479	0.1970963043936211
0.5633460300034036	0.0585430633871592	0.1974627607680601
0.3131736179454819	0.0586842312468770	0.1974089528859490
0.8131129753521266	0.0586618781485729	0.1974876171919787
0.3131860858928846	0.3088227564285342	0.1974233234399339
0.8133623311099552	0.3085745031258811	0.1972306190702847
0.0633905093431753	0.3090367083131977	0.1974440509115342
0.5630487684233840	0.3087751933588783	0.1974043595130373
0.0635291944774275	0.5588013082258249	0.1974298831603633
0.5634294893698328	0.5586073169737347	0.1976466224090933
0.3131328196342027	0.5587794484090517	0.1980135295919204
0.8131794818494024	0.5589039692276263	0.1973434210883503
0.3128599988617906	0.8091693978433079	0.1968328291816351
0.8130817515157175	0.8088385017951576	0.1975043667584436
0.0632485672472843	0.8088000144068350	0.1974571181748549
0.5633464174379396	0.8089026540276034	0.1981939831517854
0.1888357367971423	0.1832118188938264	0.2969828078223510
0.6891105198902235	0.1824572356440728	0.2969017234476271
0.4387581707184213	0.1826295589959589	0.2968716732282686
0.9388819496469952	0.1830038237181819	0.2970852207111147
0.4384191694346656	0.4341550122317653	0.2979123963026035
0.9394956874461562	0.4329146620193282	0.2968302917635239
0.1897785686475394	0.4337801272805425	0.2978907964548212
0.6894297324811988	0.4325327641880891	0.2968775952071625
0.1895611032706511	0.6824371343690877	0.2976028563467697
0.6881223042885398	0.6835848799831346	0.2979058922368411

0.4389636669591532	0.6830567789476057	0.2954944494734156
0.9393431482733406	0.6831422379494687	0.2968807730651655
0.4396239583774343	0.9326297304997908	0.2976737100435700
0.9386905581155544	0.9332132547421869	0.2970334964789334
0.1878576038317317	0.9342374088796317	0.2962703484615039
0.6884414334467083	0.9323740741925840	0.2980213586500558
0.0636901720814160	0.0582365325177605	0.3942701483157933
0.5640647520028804	0.0574024981291605	0.3943145550020326
0.3136693596483355	0.0584803935068441	0.3944516807355986
0.8143301534403178	0.0575930207441158	0.3947292233072395
0.3139043106457254	0.3064523504539362	0.3935840997020713
0.8154007280934442	0.3065178900416170	0.3941770245370050
0.0645363169097438	0.3076512978590072	0.3946631971283137
0.5652033505167948	0.3052594770058014	0.3933379565390785
0.0646062390016231	0.5579565849312749	0.3941321818958837
0.5712244850615014	0.5509176235810744	0.3932403756580083
0.3103820513378528	0.5570729528029268	0.4005144047464179
0.8169898260684849	0.5566692460744515	0.3935100118342200
0.3099718421271419	0.8120965531371230	0.3898925804513143
0.8158585739812669	0.8080840619198403	0.3937292177750772
0.0636408216199066	0.8083497852482492	0.3943717212345825
0.5651262841002626	0.8114470169348847	0.4004162845732749
0.1865870000000029	0.1853520000000017	0.0000000000000000
0.6865870000000029	0.1853520000000017	0.0000000000000000
0.4365870000000029	0.1853520000000017	0.0000000000000000
0.9365870000000029	0.1853520000000017	0.0000000000000000
0.4365870000000029	0.4353520000000017	0.0000000000000000
0.9365870000000029	0.4353520000000017	0.0000000000000000
0.1865870000000029	0.4353520000000017	0.0000000000000000
0.6865870000000029	0.4353520000000017	0.0000000000000000
0.6865870000000029	0.6853520000000017	0.0000000000000000
0.4365870000000029	0.6853520000000017	0.0000000000000000
0.9365870000000029	0.6853520000000017	0.0000000000000000
0.1865870000000029	0.6853520000000017	0.0000000000000000
0.4365870000000029	0.9353520000000017	0.0000000000000000
0.9365870000000029	0.9353520000000017	0.0000000000000000
0.1865870000000029	0.9353520000000017	0.0000000000000000
0.6865870000000029	0.9353520000000017	0.0000000000000000
0.0621859842254915	0.0597195599728078	0.0977252668087913
0.5623526983377267	0.0596892824387689	0.0977149128056934
0.3122489994387003	0.0598010805756251	0.0977099335617195

0.8123230630689724	0.0597266681269302	0.0977029714045533
0.3124279379900531	0.3096228190429094	0.0977656471785257
0.8124210016314720	0.3096023482455759	0.0977348978909450
0.0623513391601546	0.3096533048357930	0.0977428381097568
0.5623642747294976	0.3095397028385803	0.0977622802699645
0.0622692997254507	0.5596770748352577	0.0977314175759637
0.5624740882462225	0.5594688273297654	0.0977880588051082
0.3123258817398311	0.5597424846627511	0.0977492769283849
0.8123841599743603	0.5597516852463329	0.0977027517520181
0.3122410064489541	0.8098195173894710	0.0976960403823606
0.8123889602744193	0.8096899470301073	0.0977749503724786
0.0622061023348192	0.8097118522252628	0.0977715767787238
0.5623822218947530	0.8097106386471404	0.0978020565757645
0.1881814347438595	0.1838480745514182	0.1973481730874723
0.6882596472588430	0.1834796892643172	0.1973423946306023
0.4383072844877895	0.1836437953295915	0.1973877371294423
0.9381758609795783	0.1837893471910722	0.1973592320401724
0.4382895309926105	0.4335714928505757	0.1973594177149681
0.9384298090855481	0.4338279923548765	0.1973550976465197
0.1881002164272782	0.4336355863176136	0.1973106404537675
0.6885225078993689	0.4334517743201714	0.1972075816699228
0.1879065031252959	0.6837548412151296	0.1971636426521765
0.6884800615956843	0.6836855758927967	0.1974467879819661
0.4382471457357546	0.6836989522797609	0.1971965859596424
0.9382615521888503	0.6837525412350193	0.1974337996894136
0.4383560671799593	0.9340562595055916	0.1972581317779412
0.9380753603750696	0.9338178571996962	0.1973582454167594
0.1874363548542229	0.9345799526528524	0.1969592446639933
0.6884226870560584	0.9339608648754686	0.1974212326554517
0.0635095180916436	0.0585249504999896	0.2967368447141896
0.5639409753206248	0.0576046664452679	0.2972335064849778
0.3136086600635143	0.0583521559278367	0.2967248942602708
0.8139071243026818	0.0579139312415630	0.2970838998448099
0.3137416689269349	0.3078166615661510	0.2969262606558612
0.8143352228630770	0.3074266604955980	0.2966426135102614
0.0640570060713497	0.3081795553319455	0.2968883355566718
0.5642538130612144	0.3075967416548627	0.2967057597515559
0.0643661670195626	0.5579674043945256	0.2970861544081367
0.5649338374960711	0.5571274637197632	0.2965637299026309
0.3131977175346833	0.5573428779737406	0.2963347959710309
0.8144073422634791	0.5575051690357737	0.2967454674081291

0.3117694923029647	0.8103765379745274	0.2949631490657521
0.8142014262437095	0.8081617518029713	0.2970199113074155
0.0635915551598769	0.8083343088465179	0.2967372725211096
0.5649153641444433	0.8090663329396688	0.2964173322721713
0.1890215409247540	0.1826060868548104	0.3949840586921368
0.6890814837696791	0.1819775074920206	0.3948732056389226
0.4394267228145485	0.1817523186023088	0.3947571626643048
0.9393377422444855	0.1826548308081911	0.3951340993016406
0.4400206852616994	0.4251402313782304	0.3968572184298059
0.9402481902488435	0.4326108335654815	0.3948234545900666
0.1875358678111441	0.4308639712095439	0.3968604777988328
0.6932031362815747	0.4287104865438785	0.3956440293129342
0.1858300822784837	0.6863005950470427	0.3965603383759752
0.6972395610653499	0.6818696527704277	0.3968876267620941
0.4303195122393986	0.6917175774250465	0.3897199413129150
0.9402778875868728	0.6824547243736957	0.3948089447188170
0.4356896494799109	0.9364061571387061	0.3968621056717350
0.9396200450510637	0.9328438139158206	0.3950561190844725
0.1879450007821603	0.9340482017339236	0.3944024074033045
0.6915087027269005	0.9346299166357709	0.3970809389459065
0.3700778418404257	0.5798098384930175	0.4707393855715283
0.5425659413255495	0.7524956301990183	0.4705192851196608
0.5390726216332750	0.5836254721992159	0.4654849566779393
0.4636868306297925	0.6590531615497783	0.5363437968626905

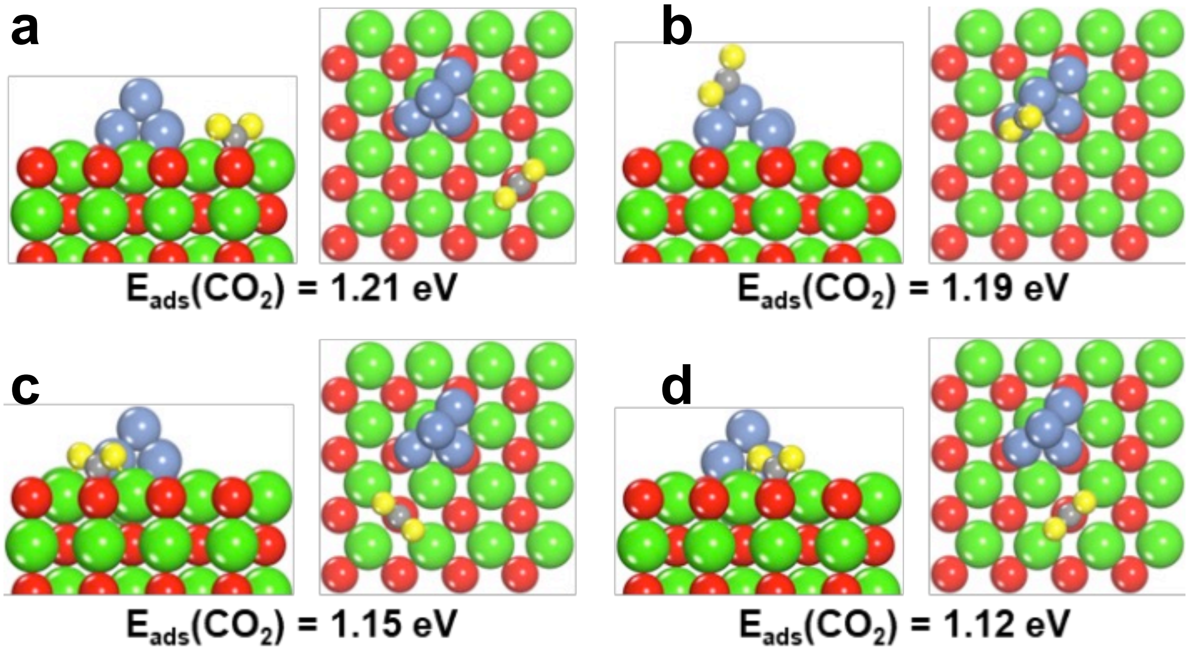

Supplementary Fig. 3 DFT calculation of CO_2 adsorption on Ni_4-CaO(100) surface. a-d Calculated structures (left: side view; right: top view) and adsorption energies of CO_2. Grey: C; yellow: O of CO_2. This notation is used throughout the paper.

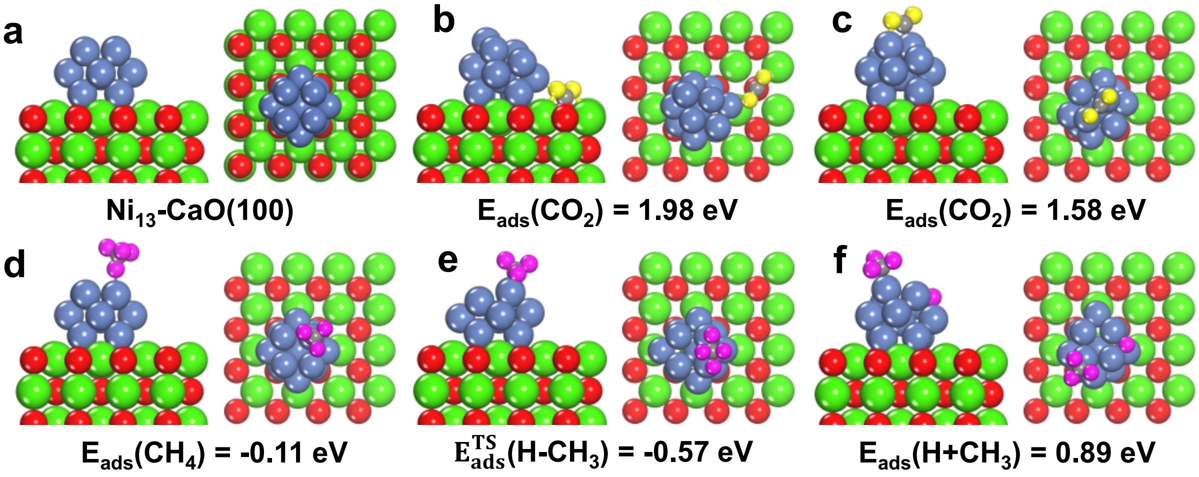

Supplementary Fig. 4 DFT calculation of CO_2 adsorption and CH_4 dissociation on the Ni_{13}-CaO (100) surface. **a** Calculated structures (left: side view; right: top view) of Ni_{13}-CaO(100) surface. **b-c** Calculated structures (left: side view; right: top view) and adsorption energies of CO_2. **d-f** Calculated structures (left: side view; right: top view) of CH_4 dissociation on the Ni_{13}-CaO(100) surface. Pink: H of CH_4. This notation is used throughout the paper.

The calculations of the CO_2 adsorption and the methane dissociation on the Ni_{13}-CaO(100) surfaces were carried out [1,2]. The results showed the same adsorption performance as those on the Ni_4-CaO that one CO_2 prefers to be adsorbed on the CaO surface by forming one C-O(CaO) bond and two (CO_2) O-Ca bonds, i.e., a carbonate-like adsorbed species (*CO_2) occurs. The corresponding calculated adsorption energy is exothermic about 1.98 eV (Supplementary Fig. 4b), stronger than that of the CO_2 on the Ni_{13} cluster (E_{ads} = 1.58 eV, Supplementary Fig. 4c). These conclusions are consistent with that in our original manuscript (the adsorption of CO_2 is exothermic by about 1.21 eV on the CaO surface and stronger than that of the CO_2 on the Ni_4 cluster (E_{ads} = 1.19 eV)). Notably, we can find that the adsorption of CO_2 on the Ni_{13}-CaO(100) surface is stronger than that on the Ni_4-CaO(100) surface, which may be caused by the richer surface and interface structures of the Ni_{13}-CaO systems for the adsorbed CO_2 to achieve more favorable interactions. In addition, we also investigated the cleavage of the C−H bond in methane at Ni_{13}-CaO(100)

(Supplementary Fig. 4d, f), and the results showed that the methane dissociation needs to overcome an energy barrier of 0.46 eV and gives an exothermic reaction energy of 1.00 eV. These results are also consistent with those in our original manuscript (the dissociation of methane needs overcome energy barrier of 0.32 eV and gives an exothermic reaction energy of 1.36 eV at Ni_4-CaO(100)).

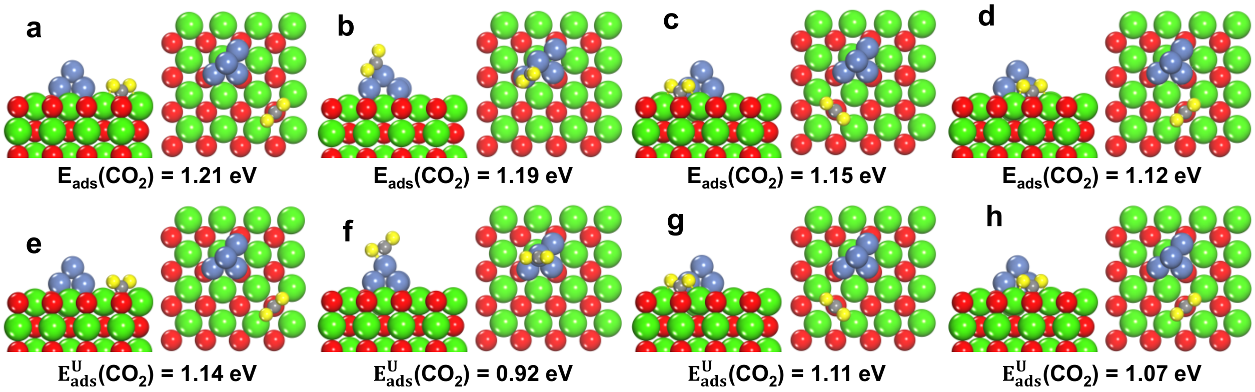

Supplementary Fig. 5 DFT calculation of CO_2 adsorption with the Hubbard U correction on Ni_4-CaO(100) surface. Calculated structures (left: side view; right: top view) and adsorption energies of CO_2 on the Ni_4-CaO(100) surface obtained without **a-d** and with **e-h** the on-site Coulomb interaction correction.

We have conducted testing calculations with the Hubbard U correction being applied to the Ni 3*d* states to better describe the atomic and electronic structures of the Ni_4-CaO(100) surfaces, where the U_d = 3.00 eV and J = 0.90 eV were used [1] respectively. The calculated results showed that the overall trend of CO_2 adsorption at different sites of the Ni_4-CaO(100) surface was consistent with that obtained without U correction (Supplementary Fig. 5). For example, the CO_2 still prefers to be adsorbed on the CaO surface by forming one C-O(CaO) bond and two (CO_2) O-Ca bonds, *i.e.*, a carbonate-like adsorbed species (*CO_2) occurs; and the corresponding adsorption energy is 1.14 eV (Supplementary Fig. 5e), stronger than that of the CO_2 on the Ni_4 cluster (0.92 eV, Supplementary Fig. 5f), both of which are also close to those obtained without U correction. Therefore, we still believe that the results we reported in our manuscript, especially those regarding the relative stabilities or reactivities.

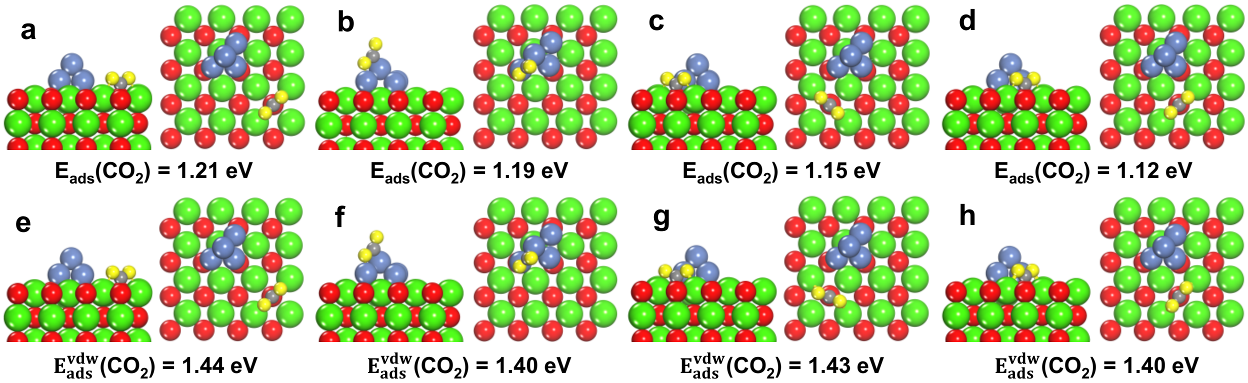

Supplementary Fig. 6 DFT calculation of CO_2 adsorption with the Grimme D2 correction on Ni_4-CaO(100) surface. Calculated structures (left: side view; right: top view) and adsorption energies of CO_2 on the Ni_4-CaO(100) surface without **a-d** and with **e-f** the Grimme D2 correction.

We have performed the testing calculations with the dispersion corrections by using the DFT-D2 method.[3,4] The calculated results showed that the adsorption energies of CO_2 on the Ni_4-CaO(100) surface increase by ~0.2 eV under the dispersion corrections, but the overall trend of CO_2 adsorption strengths at different site of the Ni_4-CaO(100) surface was largely unchanged (Supplementary Fig. 6). Also, the CO_2 prefers to be adsorbed on the CaO surface by forming one C-O (CaO) bond and two (CO_2) O-Ca bonds, i.e., a carbonate-like adsorbed species (*CO_2) occurs. Therefore, we still believe that our calculated results without considering dispersion corrections are largely reliable.

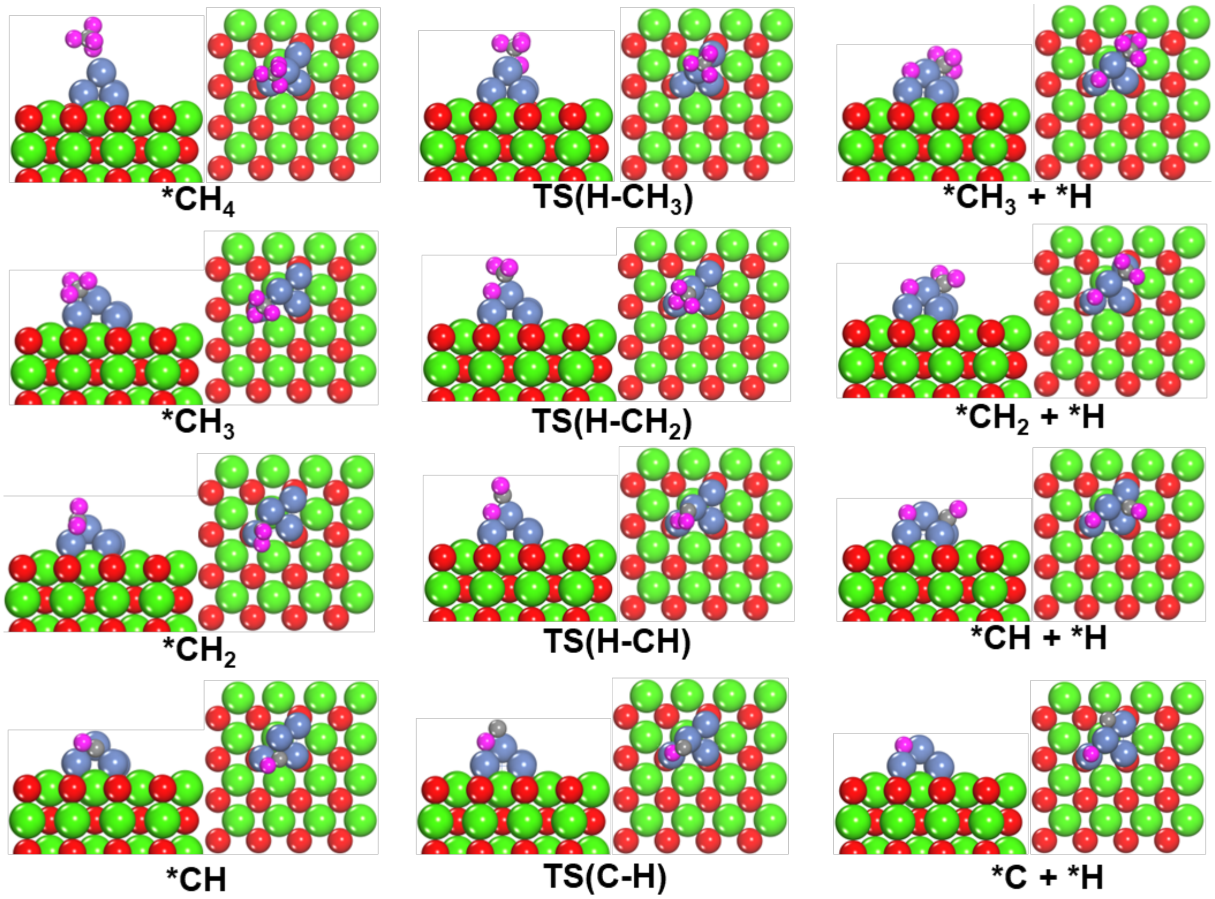

Supplementary Fig. 7 DFT calculation of CH_4 dissociation on the Ni_4-CaO (100) surface. Calculated structures (left: side view; right: top view) of CH_4 direct dissociation on the Ni_4-CaO(100) surface. Grey: C atoms; pink: H atoms. This notation is used throughout the paper.

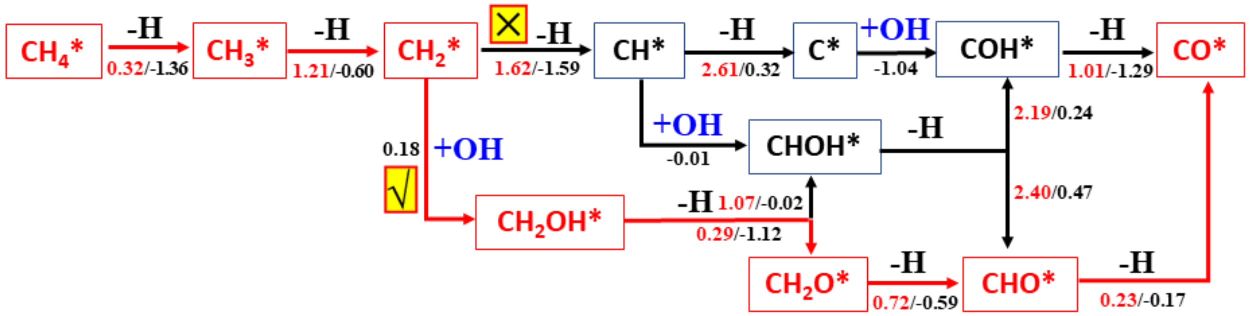

Supplementary Fig. 8 Schematics of possible reaction pathways of the CH_4 dehydrogenation with and without the assistance of *OH species on the Ni_4-CaO(100) surface. The corresponding E_a (red) and reaction energy (black) reaction for each step is also included (in eV).

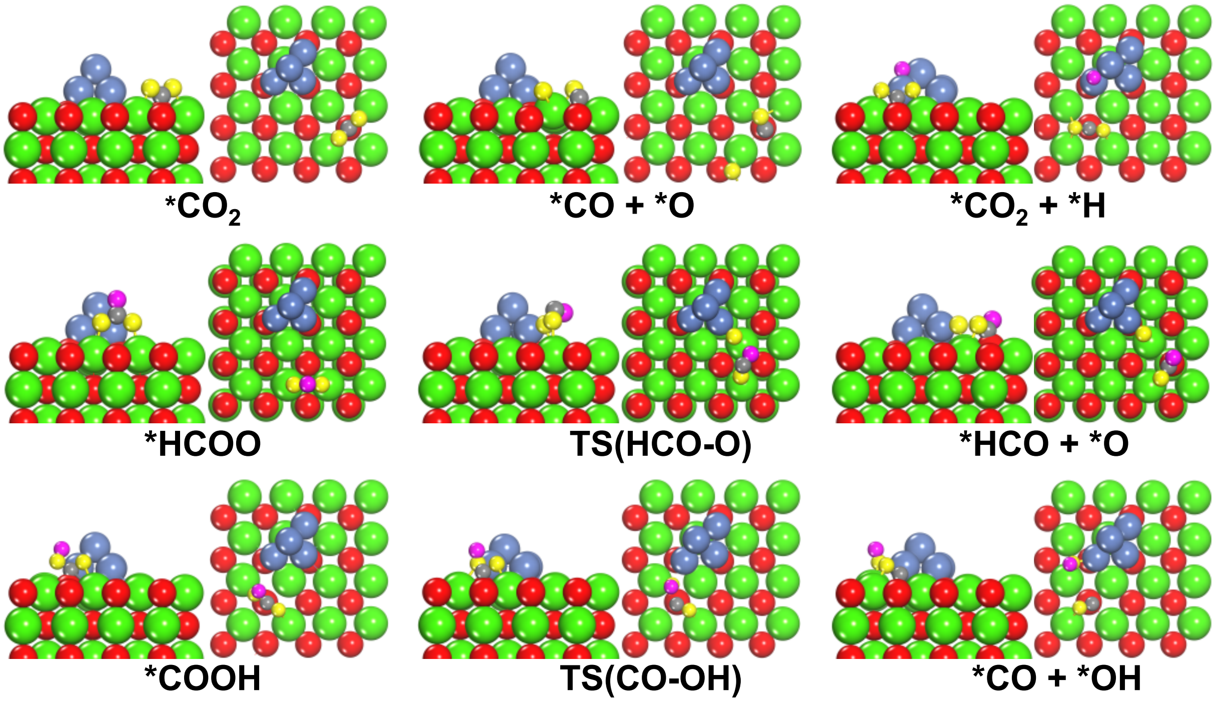

Supplementary Fig. 9 DFT calculation of H-assist CO_2 dissociation on the Ni_4-CaO (100) surface. Optimized structures (left: side view; right: top view) of H-assisted CO_2 dissociation on the Ni_4-CaO(100) surface. Yellow: O atoms of CO_2. This notation is used throughout the paper.

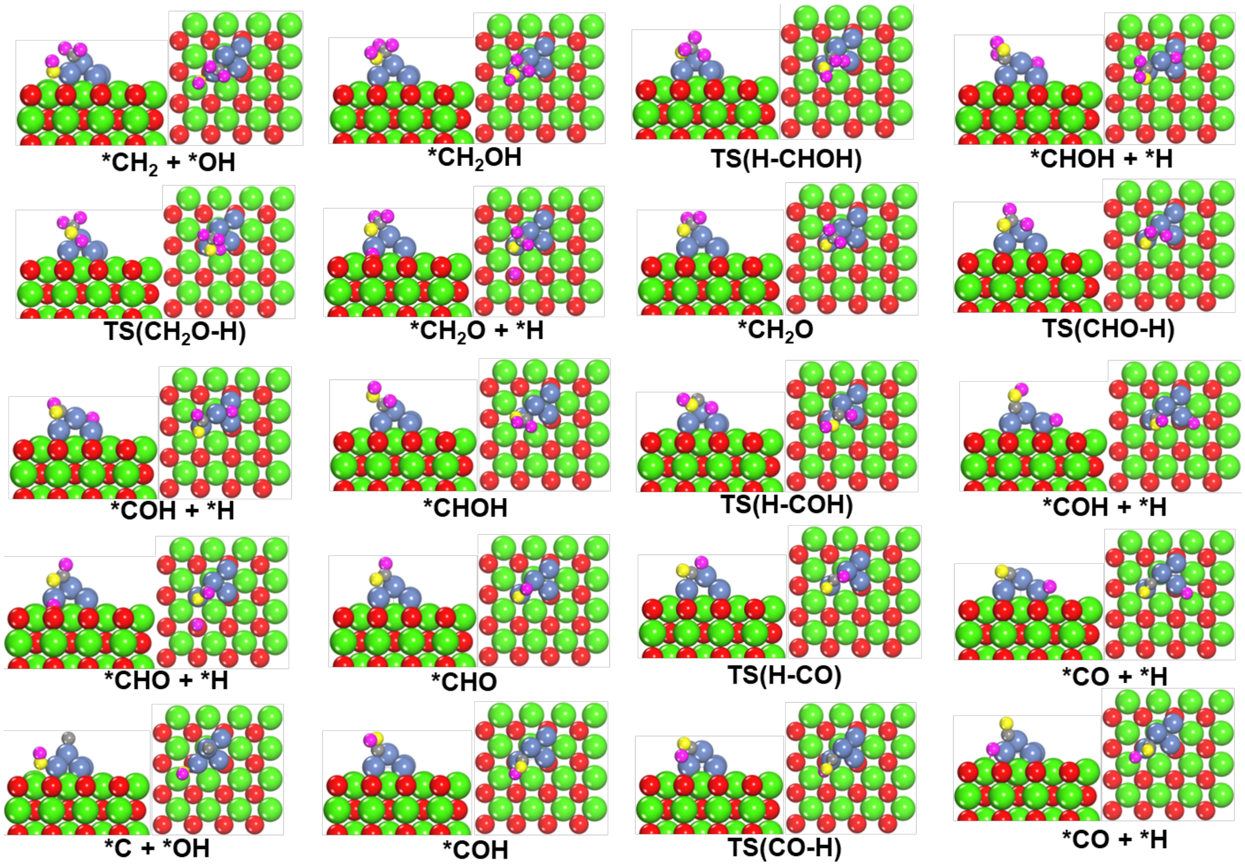

Supplementary Fig. 10 DFT calculation of OH-assist CH_4 dissociation on the Ni_4-CaO (100) surface. Calculated structures (left: side view; right: top view) of CH_4 dissociation with *OH species assisted on the Ni_4-CaO(100) surface. Grey: C atoms; pink: H atoms. This notation is used throughout the paper.

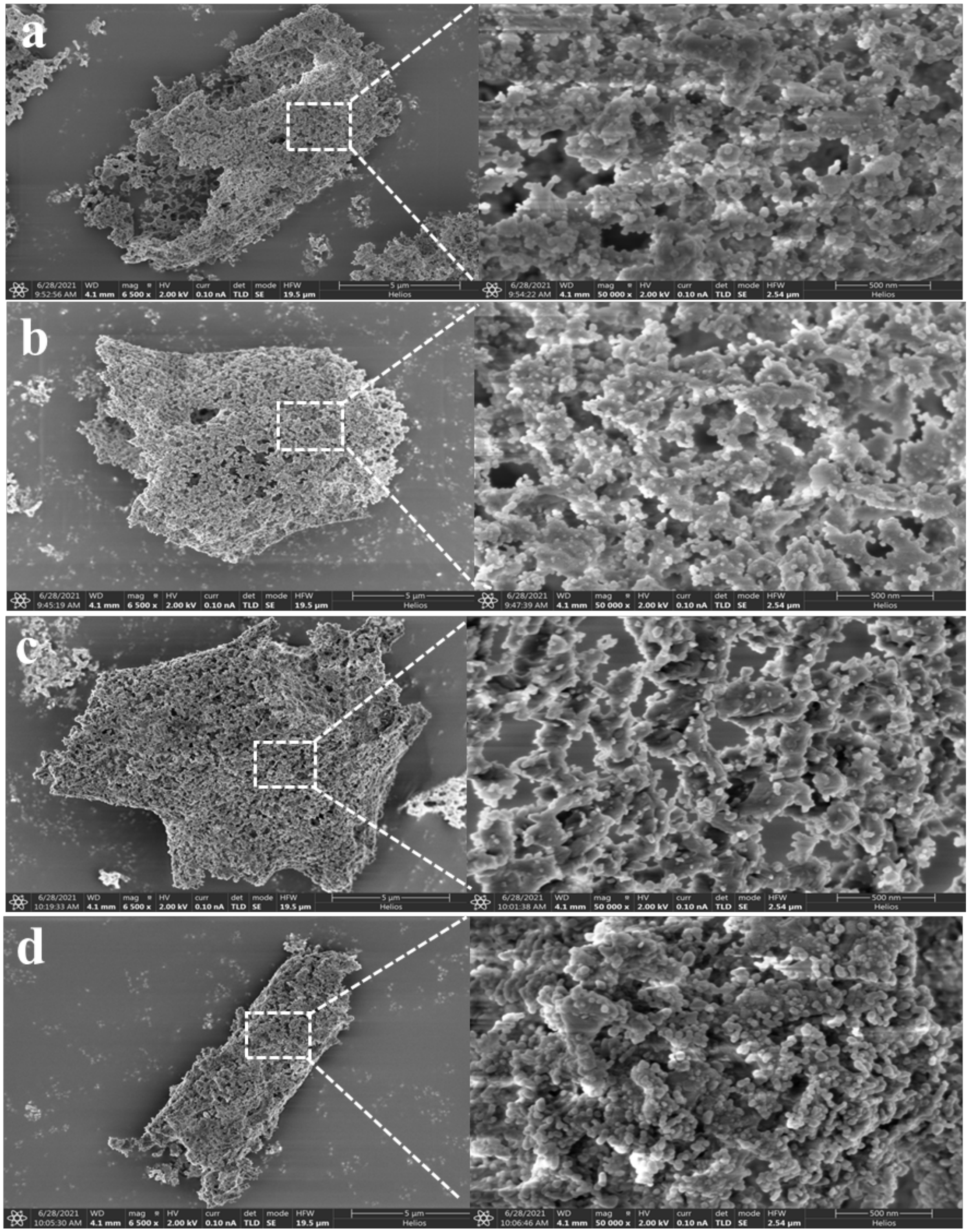

Supplementary Fig. 11 Formation of porous structures. Field emission scanning electron microscopy (FESEM) images of the prepared **a** CaO, and Ni-CaO-x DFMs of **b** Ni-CaO-2.5, **c** Ni-CaO-5 and **d** Ni-CaO-10.

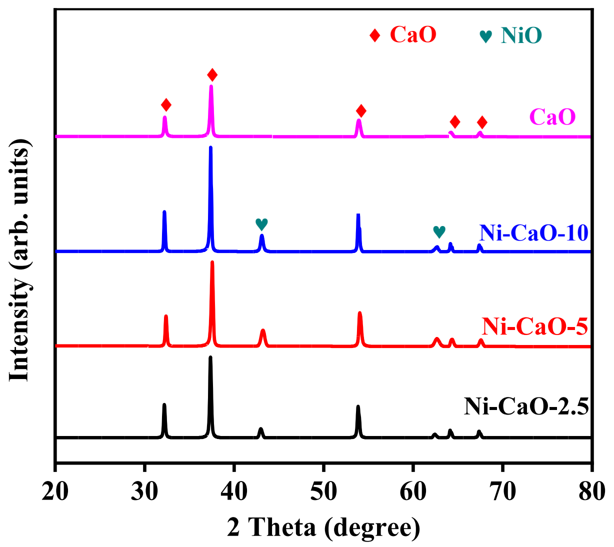

Supplementary Fig. 12 Structure characterization. XRD patterns of the as-synthesized pure CaO and Ni-CaO-x DFMs before the H_2 reduction.

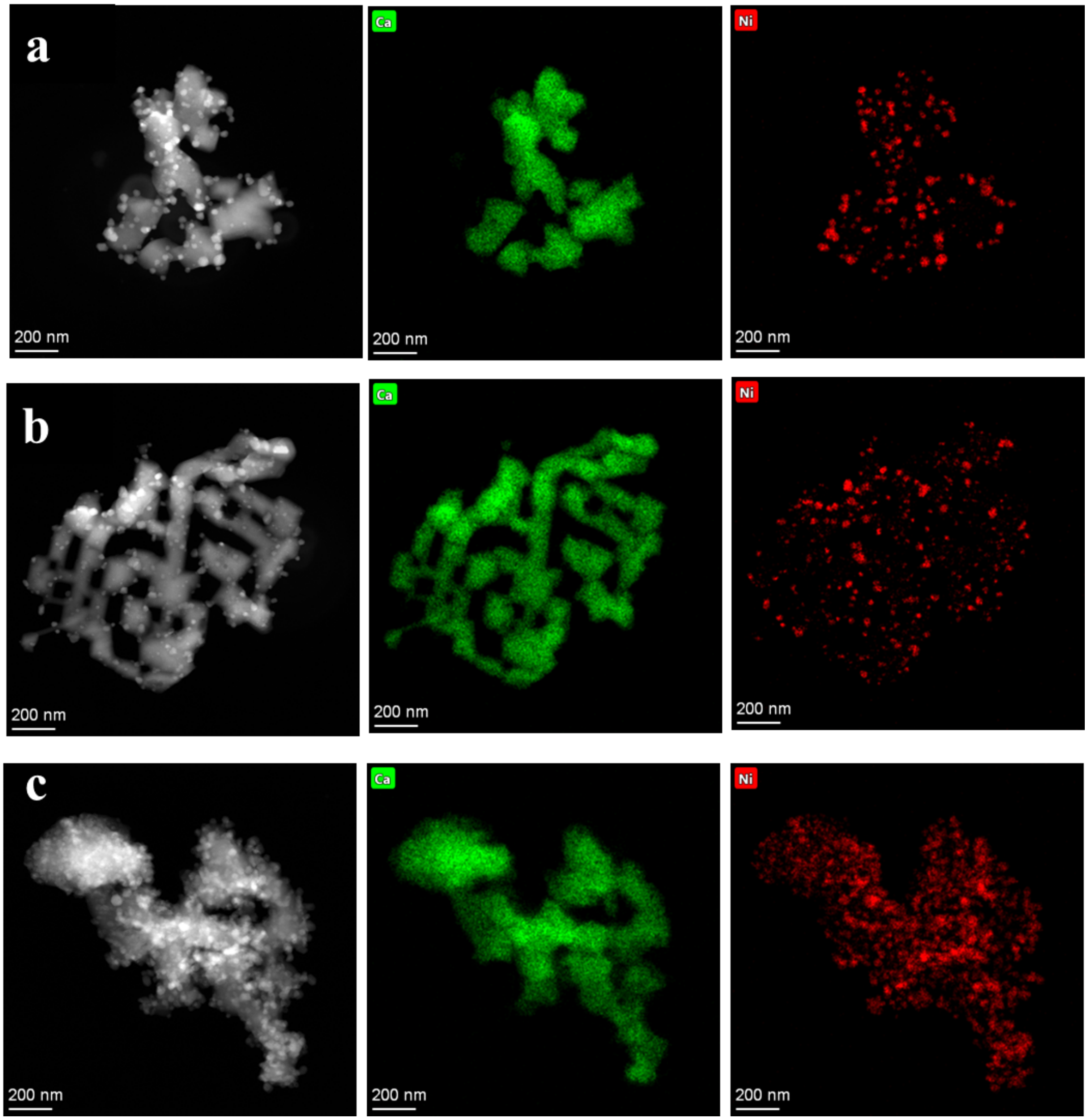

Supplementary Fig. 13 Compositional homogeneity. Elemental mappings of HADDF-TEM images for **a** Ni-CaO-2.5, **b** Ni-CaO-5, and **c** Ni-CaO-10.

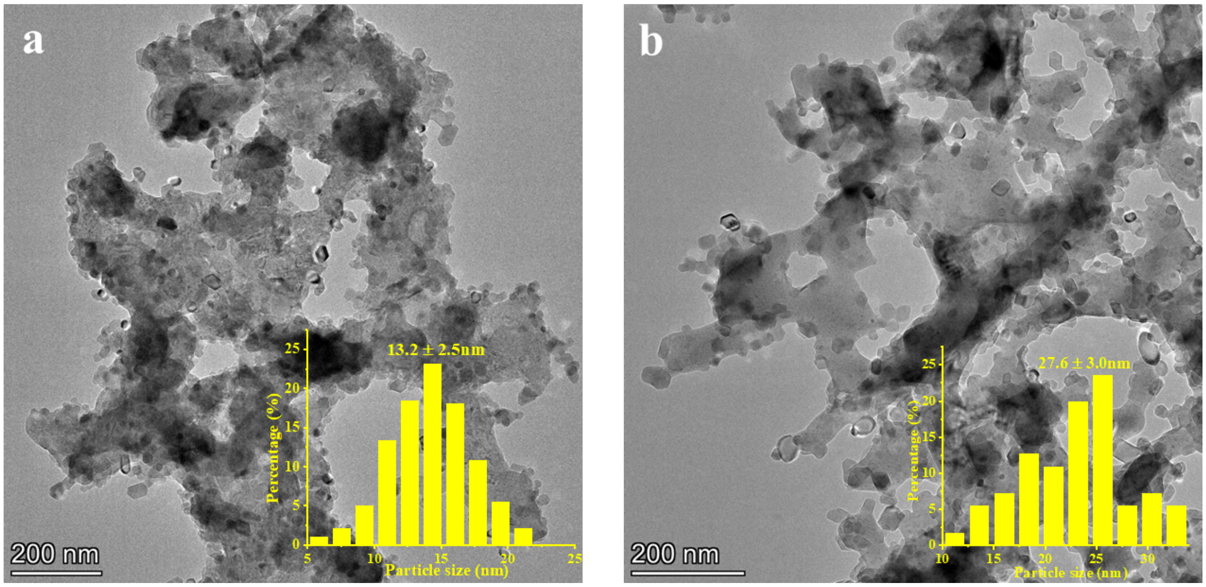

Supplementary Fig. 14 Atomic structure characterization. TEM image and size distribution of Ni nanoparticles of **a** Ni-CaO-2.5 and **b** Ni-CaO-10.

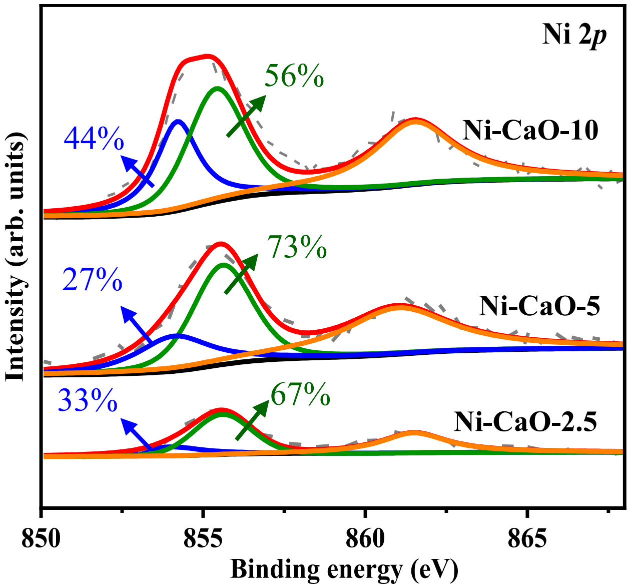

Supplementary Fig. 15 XPS characterization of Ni-CaO-x DFMs. XPS spectra of Ni 2$p_{3/2}$ where the peak at 856 eV is ascribed to the bonded Ni, and that at about 854 eV is to the free one.

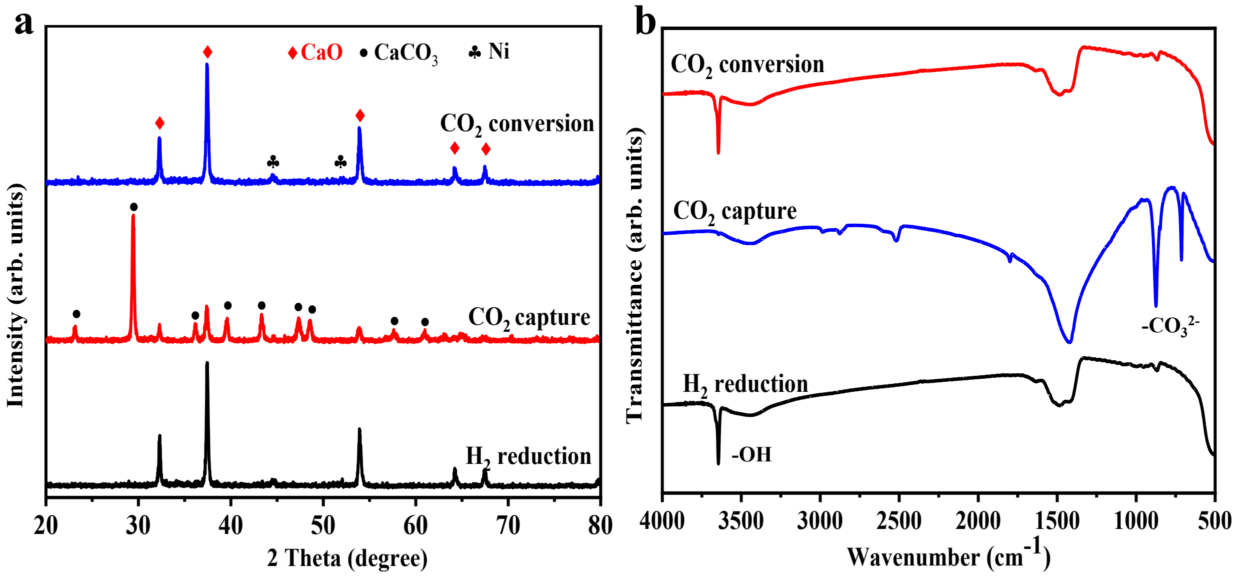

Supplementary Fig. 16 Structure characterization of iCCC on Ni-CaO-5. a XRD patterns and **b** FTIR spectrums of the Ni-CaO-5 DFM after each step of the H_2 reduction, the CO_2 capture and the *in-situ* conversion. (876 cm^{-1} and 724 cm^{-1}: vibration peak of $-CO_3^{2-}$; 3640c m^{-1}: vibration peak of -OH)

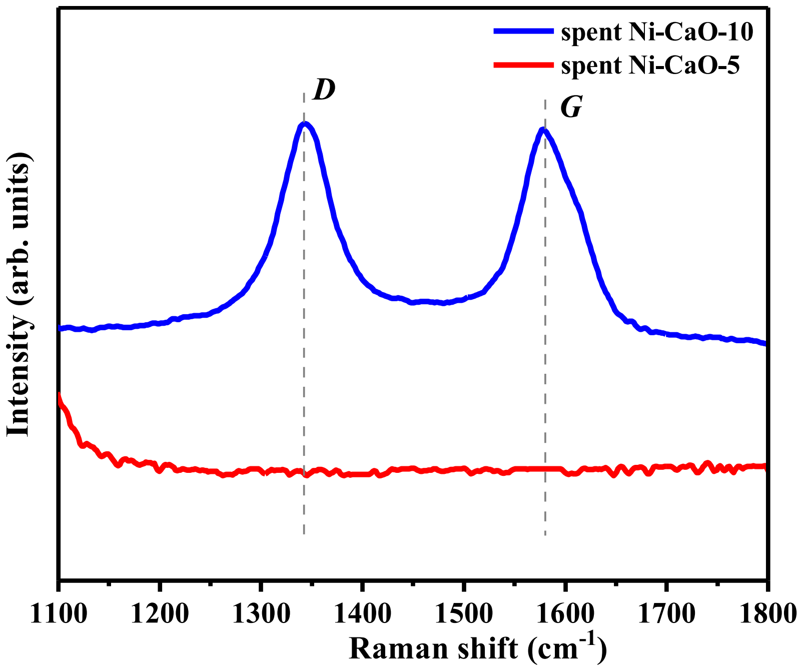

Supplementary Fig. 17 Carbon deposition analysis. Raman spectra of the carbon deposition on the spent Ni-CaO-10 (bule) and the clean surface of the spent Ni-CaO-5 (red) after 10 cycles of CaL@DRM processes.

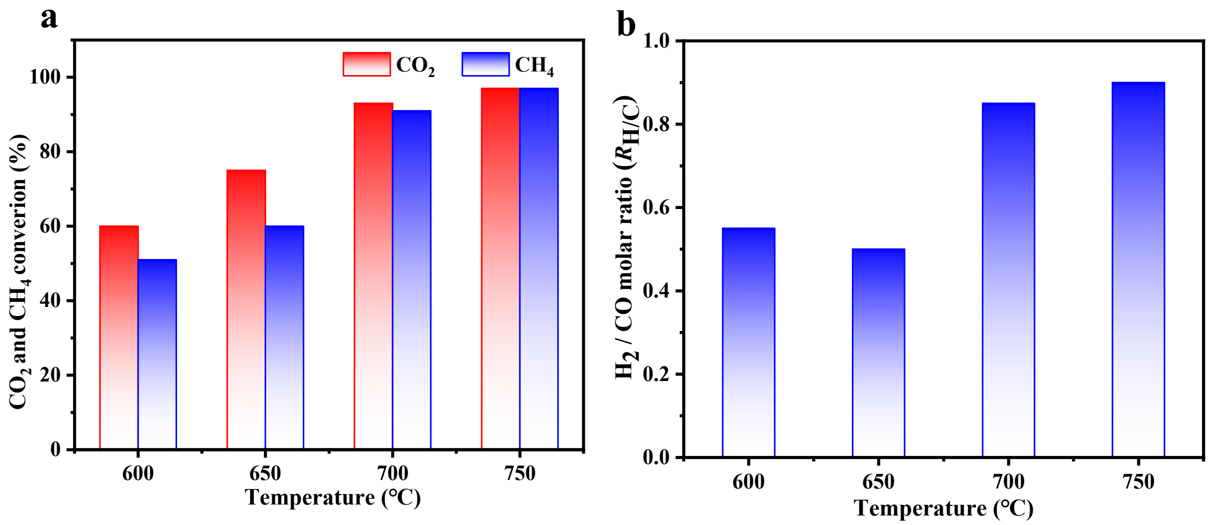

Supplementary Fig. 18 The performance of the conventional DRM on Ni-CaO-5 DFM at different temperatures. **a** the conversion of CO_2 and CH_4, **b** the molar ratio of H_2/CO in the outlet gas. The feeding gas was the mixture of 10 vol.% CO_2 and 10 vol.% CH_4 balanced with N_2.

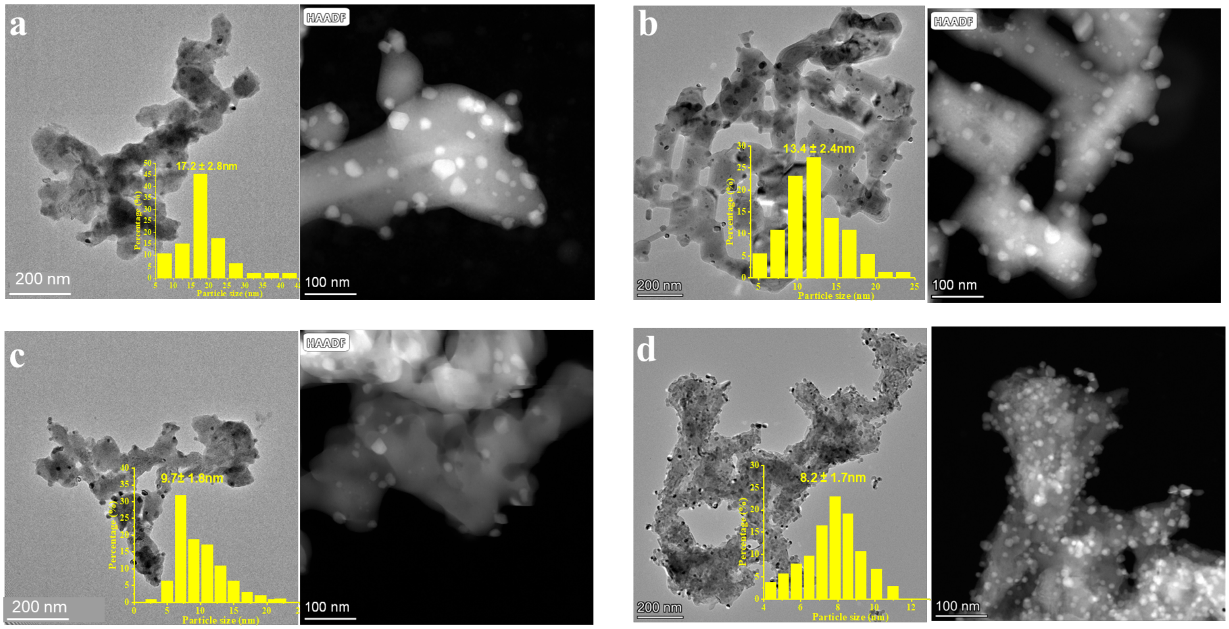

Supplementary Fig. 19 Atomic structure characterization of Ni-CaO-5(*d*). TEM image (left) and HAADF-STEM micrographs (right) of **a** Ni-CaO-5(17.3); **b** Ni-CaO-5(13.4); **c** Ni-CaO-5(9.7) and **d** Ni-CaO-5(8.2).

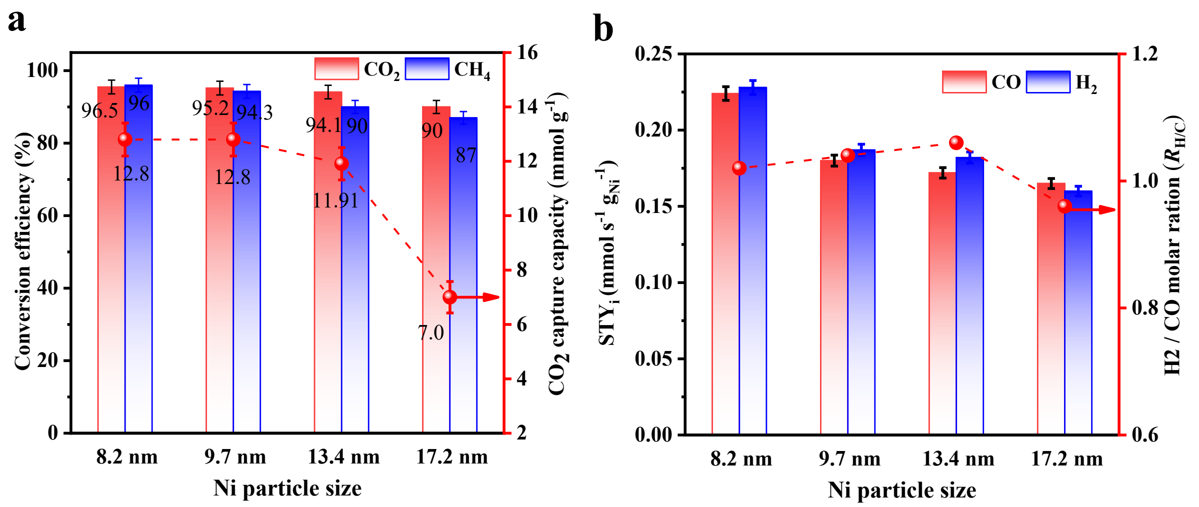

Supplementary Fig. 20 Effect of Ni particle size of Ni-CaO-5(*d*) DFM. **a** CO_2 capture capacity and *in-situ* conversion efficiency under the optimized operating conditions; **b** specific yield of H_2 and CO (average yield per gram of the loaded Ni) along with the $R_{H/C}$ in the syngas product at 650 °C. Error bars mean ± standard deviations calculated from three independent measurements.

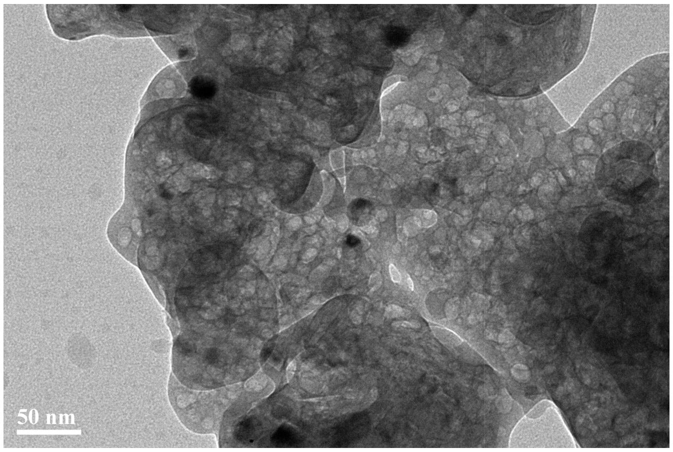

Supplementary Fig. 21 Atomic structure of Ni-CaO-5 after iCCC. TEM images of the spent Ni-CaO-5(8.2) after 10 cycles of CaL@DRM processes.

Supplementary Table 1 Characteristic properties of elemental analysis, crystallite size and porosity of Ni-CaO-x DFMs and pure CaO.

Sample	[a]Ni(wt%)	[a]Ca(wt%)	[b]D_{Ni} (nm)	Ni dispersion (%)	[c]D_{CaO} (nm)	[d]S_{BET}(m^2 g^{-1})	[e]Pore volume (cm^3 g^{-1})
Ni-CaO-10	10	63.9	13.2 ± 2.5	28	30	13	0.05
Ni-CaO-5	5	66.3	13.4 ± 2.4	35	32	20	0.07
Ni-CaO-2.5	2.5	68.9	27.6 ± 3.0	30	34	21	0.10
CaO	/	71.4	/	/	45	25	0.15

[a] measured by the inductive coupled plasma emission spectrometer (ICP)

[b] measured by size distribution of Ni nanoparticle in TEM image

[c] average crystallite size calculated by $D_p = \frac{0.94\gamma}{\beta_{1/2}\cos\theta}$, based on different diffraction peaks at 2-theta

[d] BET surface areas

[e] total pore volume at a relative pressure (P/P_0) of 0.99

Supplementary Table 2 Summary of CaO-based dual function materials and their iCCC performance.

DFMs	Reaction	Operation condition			CO_2 adsorption capacity (mol kg^{-1})	CO_2 conv. (%)	Ref.
		Adsorption	Reaction	Flow rate (ml min^{-1})			
Ni-CaO/Al_2O_3	Methanation	320 ℃ 9.5% CO_2/N_2	320 ℃ 10%H_2/N_2	100	0.31	46	5
Ru-CaO/Al_2O_3	Methanation	320 °C, 10%CO_2/N_2	320 °C, 10%H_2/N_2	17.4	0.68	96	6
Ni-CaO-Ce	RWGS	650−750 ℃ 15% CO_2/N_2	650−750 ℃ 5% H_2/N_2	100	14.1	52	7
$Fe_5Co_5Mg_{10}CaO$	RWGS	650 °C, 10% CO_2/N_2	650 °C, 100% H_2	50	9.2	90	8
Ni-(K−Ca)/γ-Al_2O_3	DER	650 ℃ 10% CO_2/N_2	650 ℃ 5%C_2H_6/N_2	30	0.99	65	9
Ni-(Na−Ca)/γ-Al_2O_3	DER	650 ℃ 10% CO_2/N_2	650 ℃ 5%C_2H_6/N_2	30	0.63	75	9
Ni-CaO catal-sorbent	DRM	700 ℃ 10%CO_2,10 % H_2O/N_2	700 ℃ 10%CH_4/N_2	40	14.8	83.8	10
Ni-Ca@Zr	DRM	720 °C, 5 %CO_2 /Ar	720 ℃ 8% CH_4/Ar	30	5	45	11
Ni-CaO-5(8.2)	DRM	650 °C, 10 %CO_2 / N_2	650 °C, 5 %CH_4 /N_2	50	12.8	96.5	**This work**

Supplementary Table 3 The performance of Ni-CaO-x DFMs with different Ni loading in the CaL@DRM processes at 650 °C.

Sample	CO_2 capacity (mmol g^{-1})	CO yield (mmol s^{-1} g_{Ni}^{-1})	H_2 yield (mmol s^{-1} g_{Ni}^{-1})	$^{a}R_{H/C}$	Carbon balance (%)	CO_2 conversion (%)	CH_4 conversion (%)
Ni-CaO-2.5	12.10	0.170	0.150	0.88	64.00	86.40	84.30
Ni-CaO-5	11.91	0.172	0.182	1.06	96.50	94.10	90.00
Ni-CaO-10	11.58	0.064	0.080	1.25	87.90	95.40	92.70
CaO	12.60	0	0	/	/	/	/

[a] $R_{H/C}$ is the molar ratio of H_2/CO in the obtained syngas.

Supplementary Table 4 Characteristic properties and activities of Ni-CaO-5 DFMs with different Ni size (*d*).

Sample Ni-CaO-5 (*d*)	[a]Ni loading (wt%)	[a]Ca loading (wt%)	Ni HAADF–STEM mean particle size (nm)	[b]Ni dispersion degree (%)	CO_2 capture capacity (mmol g^{-1})	CO_2 conversion (%)	CH_4 conversion (%)
Ni-CaO-5(17.2)	5	66.3	17.2 ± 2.8	31	7.0	90.0	87.0
Ni-CaO-5(13.4)	5	66.8	13.4 ± 2.4	35	11.9	94.1	90.0
Ni-CaO-5(9.7)	5	66.5	9.7 ± 1.8	41	12.8	95.2	94.3
Ni-CaO-5(8.2)	5	66.4	8.2 ± 2.0	45	12.8	96.5	96

[a] Ni and Ca loading determined by inductive coupled plasma emission spectrometer (ICP)

[b] Ni dispersion obtained from CO chemisorption

Supplementary Table 5. The numerical comparison of CaL@DRM iCCC processes and reported conventional DRM process in the literature.

Catalyst	Gas composition	WHSV	Temperature (℃)	CH_4 conversion (%)	CO_2 conversion (%)	H_2/CO molar ratio ($R_{H/C}$)	References
Ni-CaO-5 (8.2) DFM	**Capture step:** 10 vol.% CO_2/N_2; **Conversion step:** 5vol.% CH_4/N_2	30 L g^{-1} h^{-1} (50 ml/min)	650	96.0	96.5	1.02	**This work**
Ni-CaO catal-sorbent	**Capture step:** 10 vol.% CO_2/N_2; **Conversion step:** 10vol.% CH_4/N_2	12 L g^{-1} h^{-1} (40 ml/min)	700	/	83.8	6.53	10
Ni-Ca@Zr	**Capture step:** 5 vol.% CO_2/N_2; **Conversion step:** 8 vol.% CH_4/N_2	36 L g^{-1} h^{-1} (30 ml/min)	720	40	45	0.8	11
Ni/SiO_2-E	$CH_4/CO_2/N_2$ = 3:3:4	30 L g^{-1} h^{-1}	500	7.9	17.9	0.5	12
NiFe/Al_2O_3	CH_4/CO_2 = 1:1	12 L g^{-1} h^{-1}	550	26.6	37.8	0.67	13
0.3PdNi/MCM-41	$CH_4/CO_2/N_2$ = 1:1:3	120 L g^{-1} h^{-1}	550	37	50	0.8	14
Ni-CaO/MCM-41	CH_4/CO_2/Ar = 1:1:1	143 L g^{-1} h^{-1}	650	20	40	0.5	15
15%Ni@S-1	CH_4/CO_2 = 1:1	100 L g^{-1} h^{-1}	650	40	42	0.6	16
$Ni_3Fe_1Cu_1$-MA	$CH_4/CO_2/N_2$ = 2:2:1	432 L g^{-1} h^{-1}	650	15	35	0.5	17
Ni/MCF	CH_4/CO_2/Ar = 9:9:2	36 L g^{-1} h^{-1}	650	60	70	1.0	18

Ni/MgO	$CH_4/CO_2/Ar$ = 1:1:8	30 L g^{-1} h^{-1}	700	≥37	≥50	0.78	19
Ni/SiO_2	$CH_4/CO_2/N_2$ = 9:9:2	12 L g^{-1} h^{-1}	800	40	62	0.7	20
NiMo/MgO	$CH_4/CO_2/He$ = 1:1:8	60 L·g^{-1} h^{-1}	800	100	100	1	21

Supplementary References

1. Gueddida, S., Lebègue, S. & Badawi, M. Interaction between transition metals (Co, Ni, and Cu) systems and amorphous silica surfaces: A DFT investigation. *Appl. Surf. Sci.* **533**, 147422 (2020).
2. Gueddida, S., Badawi, M. & Lebègue, S. Grafting of iron on amorphous silica surfaces from ab initio calculations. *J. Chem. Phys.* **152**, 214706 (2020).
3. Bučko, T., Hafner, J., Lebègue, S. & Ángyán, J.G. Improved description of the structure of molecular and layered crystals: Ab initio DFT calculations with van der waals corrections. *J. Phys. Chem. A* **114**, 11814-11824 (2010).
4. Grimme, S. Semiempirical GGA-type density functional constructed with a long-range dispersion correction. *J. Comput. Chem.* **27**, 1787-1799 (2006).
5. Chai, K.H., Leong, L.K., Wong, D.S.H., Tsai, D.H. & Sethupathi, S. Effect of CO_2 adsorbents on the Ni-based dual-function materials for CO_2 capturing and in situ methanation. *J. Chin. Chem. Soc.* **67**, 998-1008 (2020).
6. Arellano-Treviño, M.A., He, Z., Libby, M.C. & Farrauto, R.J. Catalysts and adsorbents for CO_2 capture and conversion with dual function materials: Limitations of Ni-containing DFMs for flue gas applications. *J.* CO_2 *Utiliz.* **31**, 143-151 (2019).
7. Sun, H. et al. Dual functional catalytic materials of Ni over Ce-modified CaO sorbents for integrated CO_2 capture and conversion. *Appl. Catal. B Environ.* **244**, 63-75 (2019).
8. Shao, B. et al. Heterojunction-redox catalysts of $Fe_xCo_yMg_{10}CaO$ for high-temperature CO_2 capture and in situ conversion in the context of green manufacturing. *Energy Environ. Sci.* **14**, 2291-2301 (2021).
9. Al-Mamoori, A., Rownaghi, A.A. & Rezaei, F. Combined capture and utilization of CO_2 for syngas production over dual-function materials. *ACS Sustainable Chem. Eng.* **6**, 13551-13561 (2018).
10. Jo, S.B. et al. CO_2 green technologies in CO_2 capture and direct utilization processes: methanation, reverse water-gas shift, and dry reforming of methane. *Sustain. Energy Fuels* **4**, 5543-5549 (2020).
11. Hu, J., Hongmanorom, P., Galvita, V.V., Li, Z. & Kawi, S. Bifunctional Ni-Ca based material for integrated CO_2 capture and conversion via calcium-looping dry reforming. *Appl. Catal. B Environ.* **284**, 119734 (2021).
12. Wang, F., Han, K., Xu, L., Yu, H. & Shi, W. Ni/SiO_2 catalyst prepared by strong

electrostatic adsorption for a low-temperature methane dry reforming reaction. *Ind. Eng. Chem. Res.* **60**, 3324-3333 (2021).
13. Song, Z. et al. Improved effect of Fe on the stable NiFe/Al_2O_3 catalyst in low-temperature dry reforming of methane. Ind. Eng. Chem. Res. **59**, 17250-17258 (2020).
14. Damyanova, S. et al. MCM-41 supported PdNi catalysts for dry reforming of methane. Appl.Catal. B Environ. **92**, 250-261 (2009).
15. Wu, P. et al. Cooperation of Ni and CaO at interface for CO_2 reforming of CH_4: A combined theoretical and experimental study. ACS Catal. **9**, 10060-10069 (2019).
16. Liu, Y. et al. Embedding high loading and uniform Ni nanoparticles into silicalite-1 zeolite for dry reforming of methane. Appl.Catal. B Environ. **307**, 121202 (2022).
17. Jin, F. et al. Stable trimetallic NiFeCu Catalysts with high carbon resistance for dry reforming of methane. ChemPlusChem **85**, 1120-1128 (2020).
18. Daoura, O. et al. Mesocellular silica foam-based Ni catalysts for dry reforming of CH_4 (by CO_2). J. CO_2 Utiliz. **24**, 112-119 (2018).
19. Cheng, F., Duan, X. & Xie, K. Dry Reforming of CH_4 /CO_2 by stable Ni nanocrystals on porous single-crystalline MgO monoliths at reduced temperature. Angew. Chem. Int. Ed. Engl. (2021).
20. Han, J.W., Park, J.S., Choi, M.S. & Lee, H. Uncoupling the size and support effects of Ni catalysts for dry reforming of methane. Appl.Catal. B Environ. **203**, 625-632 (2017).
21. Song, Y. et al. Dry reforming of methane by stable Ni–Mo nanocatalysts on single-crystalline MgO. Science **367**, 777-781 (2020).
